# Improving Access to Specialty Care for Rural Children Using Enhanced Hearing Screening and Specialty Telehealth Follow-Up in Rural Kentucky Schools: Protocol for a Hybrid Effectiveness-Implementation Stepped Wedge, Cluster-Randomized Controlled Trial (Appalachian STAR Trial)

**DOI:** 10.2196/77630

**Published:** 2025-08-26

**Authors:** Tarika Srinivasan, Samantha Kleindienst Robler, Elizabeth Turner, Alyssa Platt, David Arthur, Janet Prvu Bettger, Hannah Lane, Marissa Schuh Gebert, Shayu Deshpande, Nancy Schoenberg, Matthew L Bush, Susan D Emmett

**Affiliations:** 1 Harvard Medical School Harvard University Boston United States; 2 Center for Hearing Health Access University of Arkansas for Medical Sciences Little Rock, AR United States; 3 Department of Otolaryngology-Head and Neck Surgery College of Medicine University of Arkansas for Medical Sciences Little Rock, AR United States; 4 Duke Global Health Institute Duke University Durham, NC United States; 5 Department of Biostatistics and Bioinformatics Duke University Durham, NC United States; 6 Department of Population Health Sciences Duke University Durham United States; 7 Department of Otolaryngology-Head and Neck Surgery University of Kentucky Lexington, KY United States; 8 Department of Behavioral Science University of Kentucky Lexington United States; 9 Department of Epidemiology Fay W Boozman College of Public Health University of Arkansas for Medical Sciences Little Rock, AR United States

**Keywords:** hearing loss, health disparities, specialty care, rural health, telehealth, telemedicine, school, children, hearing screening, randomized controlled trial, cluster-randomized trial, stepped wedge, implementation

## Abstract

**Background:**

Rural populations are disproportionately affected by preventable childhood hearing loss, which is associated with speech and language delays, impaired social development, and decreased educational attainment. Rural schools are critical access points for preventive health screenings such as hearing screening, but variable screening implementation, loss to follow-up, and scarcity of specialists in rural areas diminish program effectiveness. We seek to implement a school-based telehealth intervention to increase access to specialty hearing care for children in rural Kentucky.

**Objective:**

The Appalachian Specialty Telemedicine Access for Referrals (STAR) trial will assess effectiveness and implementation of the novel, evidence-based STAR model, consisting of 3 core components: (1) enhanced hearing screening; (2) specialty telehealth follow-up; and (3) streamlined communication between schools, health care providers, and parents and caregivers.

**Methods:**

Adaptation of the STAR model for rural Kentucky will occur in the first 2 years, followed by a phased rollout of the intervention using a stepped wedge, cluster-randomized design among kindergartners enrolled in approximately 63 schools in 14 counties of rural Kentucky. School districts were identified based on scientific and community input, as well as geographic proximity to state-run clinics, which provide audiology evaluation free of charge. School districts were randomized into 2 sequences using constrained randomization to balance baseline covariates, such as kindergarten enrollment and number screened. This hybrid type 1 effectiveness-implementation trial will evaluate effectiveness of the STAR model compared with usual hearing screening and usual follow-up process using an intention-to-treat approach with generalized estimating equations. Barriers and facilitators to implementation of the intervention will be identified using a mixed methods approach. The primary effectiveness outcomes are the (1) proportion of kindergarteners screened and (2) proportion of referred kindergarteners who receive specialty follow-up within 60 days of screening. Implementation outcomes include assessment of factors affecting successful integration of the STAR model. Iterative adaptation of the intervention will be performed at prespecified time points to maximize implementation outcomes.

**Results:**

The trial began in September 2022 and is expected to conclude in May 2026. Final data analysis is planned to begin in June 2026, and publication of results is expected in 2027.

**Conclusions:**

The STAR model addresses issues related to identification of hearing loss, loss to follow-up from screening, and access to specialty care in rural Kentucky. Effectiveness outcomes may inform future policy for school hearing screening, including adoption of evidence-based protocols to address preventable childhood hearing loss and integration of school-based specialty telehealth follow-up to improve follow-up. Implementation aims may maximize the STAR model’s adaptability and overall fit. Community input and systematic adaptation will ensure consideration of unique needs and priorities of rural Kentucky counties. If successful, the STAR model could be scaled across rural America and applied to other preventable child health conditions.

**Trial Registration:**

ClinicalTrials.gov NCT05513833; https://clinicaltrials.gov/study/NCT05513833

**International Registered Report Identifier (IRRID):**

DERR1-10.2196/77630

## Introduction

### Background

Individuals living in rural communities experience worse health outcomes beginning early in life, making health interventions targeting young children essential to improve health in rural communities [1-5]. Limited access to health care in rural areas is a major contributor to these differences [6-8]. Ear and hearing health constitutes a prime example of how geography can affect health outcomes. Untreated childhood hearing loss has serious long-term consequences, including speech and language delays, worse academic performance, lower literacy, impaired social skills, and reduced higher education and employment opportunities [9-12]. Children with even mild hearing loss have lower quality of life and worse behavioral outcomes than peers with normal hearing [13,14]. The World Health Organization (WHO) estimates that up to 60% of childhood hearing loss is due to preventable causes, such as untreated otitis media, and this estimate rises to 75% in resource-constrained settings such as rural communities [9,15]. Low socioeconomic status, lack of insurance, and geographic barriers to accessing specialty care are associated with poorer outcomes in rural patients with ear or hearing conditions [16-19]. As such, childhood hearing loss and its predisposing conditions disproportionately affect rural and other underserved populations and constitute a critical intervention point to prevent more intractable long-term health and social disadvantage [16,20-22].

Schools serve as an essential access point for health screening and preventive interventions for rural American children. Over 95% of children attend school during critical years of social and intellectual development, making schools an ideal location for preventive health screenings recommended by the American Academy of Pediatrics [23-25]. School nurses and teachers engage daily with rural children, strengthening their potential as key implementation partners for preventive health programs. In the United States, school-based hearing screening programs are designed to identify cases of acquired or later-onset childhood hearing loss [26]. Although school screening can support early evaluation and treatment of childhood hearing loss and may be cost-effective [27], challenges remain with implementation, such as a lack of standardized screening protocols and frequent loss to follow-up. Further, the necessary diagnosis, treatment, and monitoring provided by audiologists and otolaryngologists are not available in primary care [28,29].

**Figure 1:**
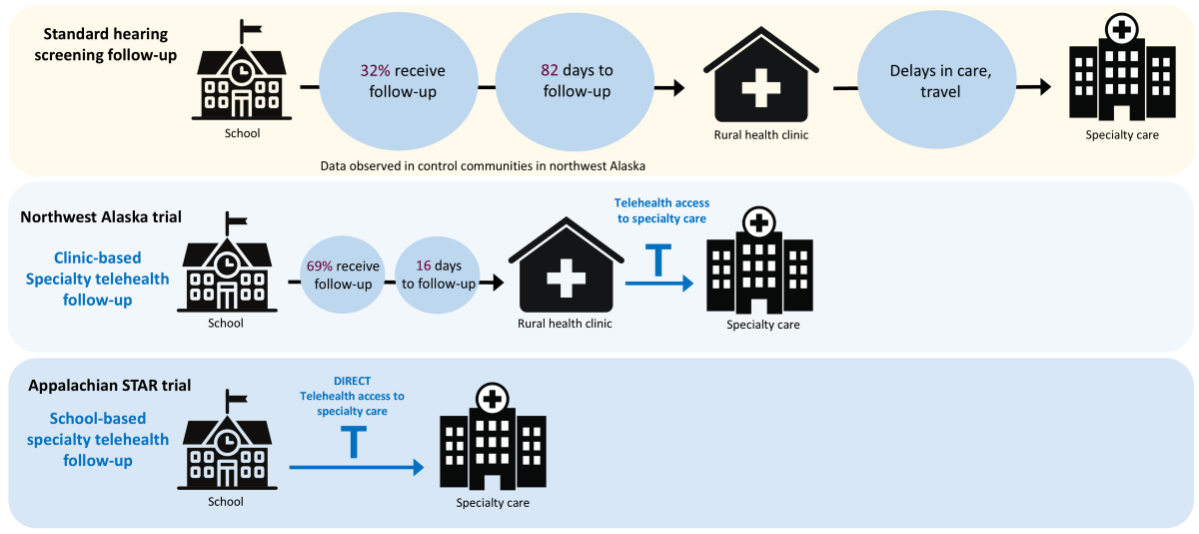
Comparison of follow-up pathways following school hearing screening, with data for the first 2 rows provided from a trial in northwest Alaska [35]. STAR: Specialty Telemedicine Access for Referrals.

**Figure 2:**
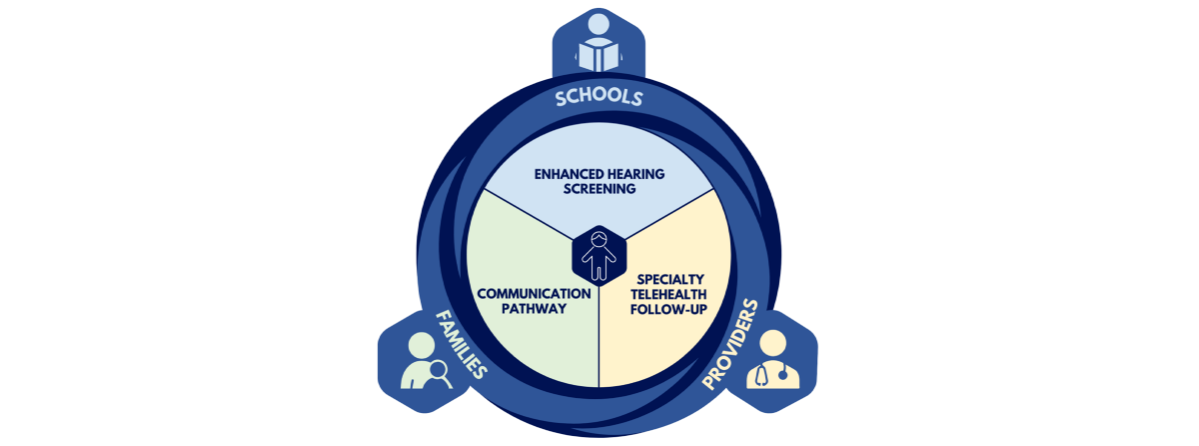
Core components of the Specialty Telemedicine Access for Referrals (STAR) model.

The Appalachian Specialty Telemedicine Access for Referrals (STAR) trial seeks to combine the potential of the school as the primary location for preventive health services with telehealth specialty follow-up to improve access to specialty care in rural areas. Telehealth has increasingly been used within innovative programs designed to enable access to specialty care among rural populations with a dearth of health care resources [30-32]. Our group previously conducted a randomized trial in rural northwest Alaska which was, to our knowledge, the first to integrate telehealth into an intervention to address loss to follow-up from screening and provide better access to specialists in rural areas [33]. This Patient-Centered Outcomes Research Institute (PCORI)–funded trial used the existing telehealth infrastructure in rural clinics within the Tribal health system of rural Alaska for follow-up from school hearing screening (Figure 1). Several key findings from that randomized trial have contributed to the development of the STAR model. First, although infection-related hearing loss is common in rural communities and other countries have begun to incorporate tympanometry into national screening protocols [34], current screening protocols in the United States do not yet include a middle ear assessment that is essential for detecting infection-related hearing loss. Our trial found that the addition of tympanometry significantly increased sensitivity compared with usual school screening [33]. Second, telehealth specialty follow-up more than doubled the likelihood of a child receiving follow-up care, and that care happened over 17 times faster on average with telehealth than with usual follow-up. Third, based on qualitative interviews with stakeholders, we found that success of the telehealth intervention varied depending on clinic capacity, personnel and ownership, awareness, and communication between schools and clinics [35], and a key recommendation was to provide telehealth capacity directly within the school setting. As a result, the STAR model incorporates (1) enhanced hearing screening with tympanometry; (2) school-based specialty telehealth follow-up; and (3) streamlined communication between schools, health care providers, and families (Figure 2).

In the Appalachian STAR trial, we seek to address the implementation factors identified in our prior trial and translate the STAR model for application in a broader rural context. Prior work by members of our group has established that residents of rural Appalachian Kentucky communities experience delays in access to ear and hearing specialty care and have overall worse ear and hearing outcomes [16,18,20]. Based on this prior work and community input, we selected the rural Appalachian communities of eastern Kentucky as the setting for translation of the STAR model. We adapted the evidence-based STAR model initially designed in rural indigenous Alaskan communities to meet the unique needs and cultural context of these rural Kentucky populations by using the systematic evidence-based ADAPT-ITT framework [36]. The ADAPT-ITT model was selected to guide this process because it is a method to facilitate adaptation of existing evidence-based interventions for novel situations and environments. We also engage community members and stakeholders in the implementation evaluation, forming the basis for community involvement in sustainable implementation of the STAR model in Appalachia and expansion into other regions of rural America.

### Aims

The effectiveness and implementation of the STAR model are being assessed via 3 distinct aims. Aim 1 used community engagement strategies to inform the adaptation of the STAR model to meet the unique needs of communities and schools in rural Kentucky prior to implementation using the ADAPT-ITT framework [36]. Aim 2 evaluates effectiveness of the STAR model via a nested stepped wedge, cluster-randomized trial in 14 counties (approximately 63 schools) in rural Kentucky, comparing the STAR model with usual hearing screening and follow-up processes. Finally, Aim 3 assesses implementation factors and outcomes in rural Kentucky schools to drive further adaptations during the trial and inform overall scalability of the STAR model.

### Hypotheses

We conservatively hypothesize that the probability of being screened for hearing loss will be greater for children in schools that receive enhanced hearing screening via the STAR intervention compared with those with usual hearing screening. We further hypothesize that the probability of specialty follow-up within 60 days of screening for children who are referred will be greater in schools that have implemented both enhanced hearing screening and the specialty telehealth follow-up compared with those that have only implemented enhanced screening [37].

## Methods

### Study Setting

**Figure 3:**
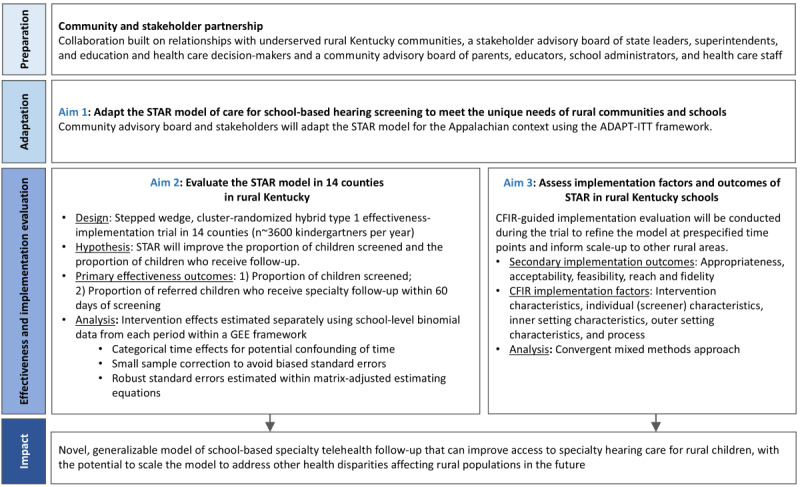
Schematic of the study phases for the Appalachian Specialty Telemedicine Access for Referrals (STAR) trial. CFIR: Consolidated Framework for Implementation Research; GEE: generalized estimating equation.

**Figure 4:**
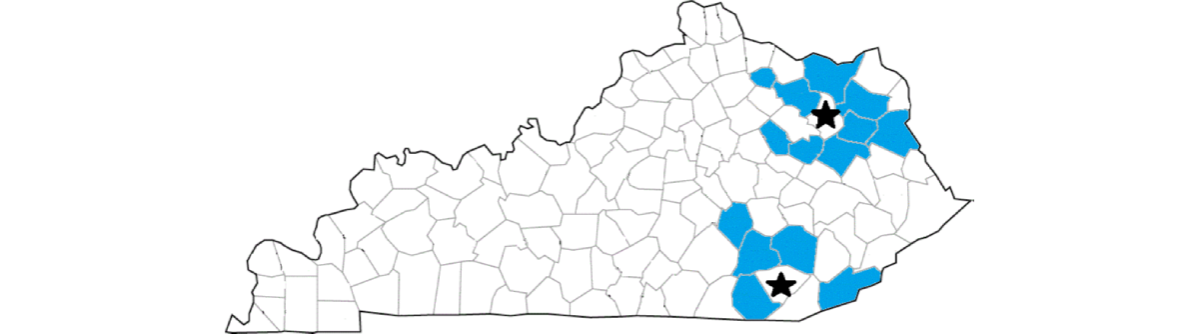
Map of Kentucky counties participating in the Appalachian Specialty Telemedicine Access for Referrals (STAR) trial (indicated in blue), including counties with state-run specialty clinics (denoted with a star).

The Appalachian STAR trial assesses effectiveness among kindergartners (~3600/year) attending 63 elementary schools in 14 participating counties in rural eastern Kentucky. This region was chosen based on feedback from scientific and community stakeholders with a long-standing partnership in this region (Figure 3). Participating counties have long been classified as persistent poverty counties and are among the poorest in the United States, with most children considered underserved, rural, and socioeconomically disadvantaged. Like many rural areas of the United States, the region has limited access to specialty care. Hearing screening is required for all children at entry into elementary schools in Kentucky [38] but is currently variably implemented across school districts [39]. Children who receive referrals from school hearing screening programs can undergo diagnostic audiology evaluation at clinics administered by the Office of Children with Special Healthcare Needs (OCSHCN) free of charge, though the program is currently not well-utilized. After receiving community input, participating counties were selected based on close proximity to 2 eastern Kentucky counties with an OCSHCN clinic. Personnel at these clinics will facilitate the specialty telehealth follow-up component of the STAR intervention (Figure 4).

### Stakeholder Feedback

This study builds on strong community engagement and stakeholder partnership. The scientific team has been meeting regularly since study inception with a community advisory board (CAB) comprised of parents, educators, school administrators, and health care providers, as well as a stakeholder advisory board (SAB) comprised of superintendents of the participating school districts and key representatives from the Kentucky Department of Education, OCSHCN, Cabinet of Health and Family Services, and Department of Medicaid Services. In Aim 1, we engaged participating communities and incorporated the preferences and perspectives of parents, providers, educators, school administrators, the CAB, and other stakeholders to adapt the STAR model for rural Kentucky using the ADAPT-ITT framework [36]. During and after STAR model deployment in the stepped wedge cluster-randomized trial (Aim 2), stakeholders provide feedback on their experiences with the STAR model. This feedback is being used to develop iterative refinements at predefined time points during the trial and ensures the design of the STAR model is informed by community engagement (Aim 3). Refinements include implementation-related elements of the model, such as how informed consent for telehealth follow-up is obtained and how training materials are designed or delivered. Changes are not being made to the core components of the STAR model. Implementation is being systematically evaluated to assess adoption, acceptability, fidelity, and implementation costs [40,41]. 

### Study Design

**Figure 5:**
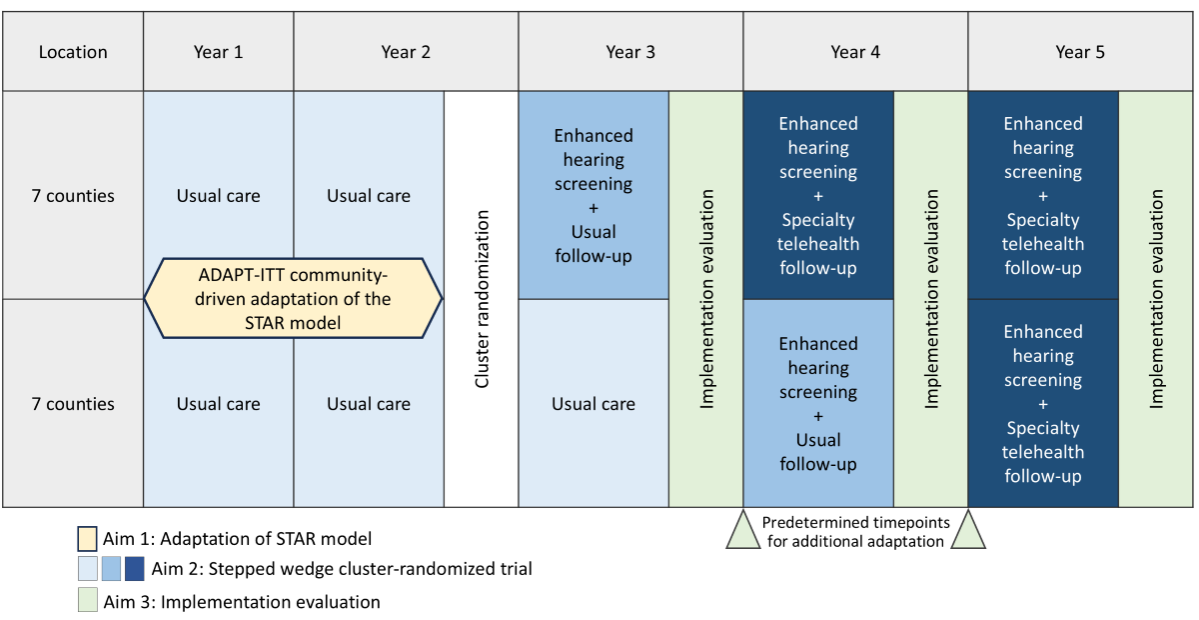
Appalachian Specialty Telemedicine Access for Referrals (STAR) trial timeline.

The Appalachian STAR trial began with adaptation of the STAR model for the local context informed by feedback from community partners in rural eastern Kentucky counties (Aim 1; Figure 5). A hybrid type I design is being used to test the effectiveness of the STAR intervention on hearing screening and specialty follow-up (Aim 2). The study is also evaluating the implementation of the STAR intervention using both qualitative and quantitative methods (Aim 3). All schools in participating counties continued with hearing screening and referral according to the current practices for that county for the first 2 years to determine baseline screening and follow-up. This is followed by a phased rollout of the STAR model (ie, first enhanced hearing screening then specialty telehealth follow-up), with an implementation evaluation ongoing throughout the trial.

**Figure 6:**
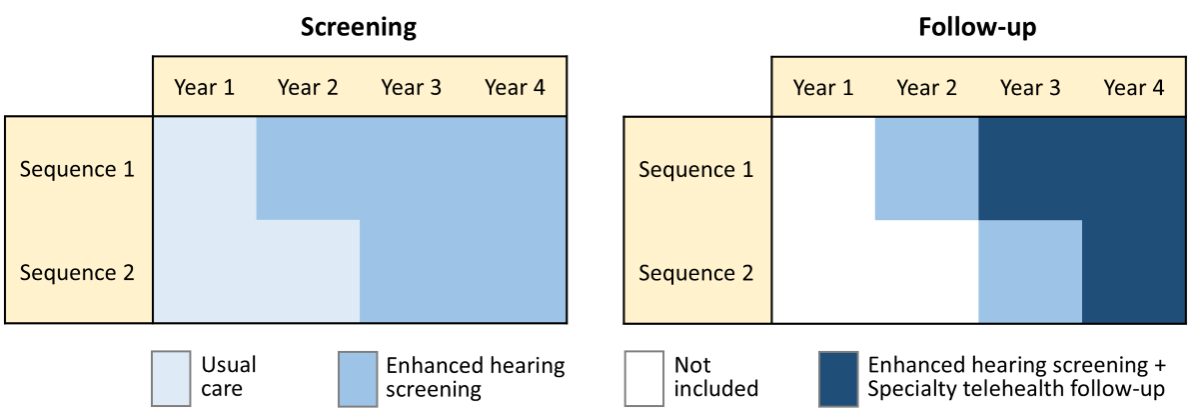
Graphic of complete and incomplete stepped wedge trial designs for the screening and follow-up outcomes.

The Appalachian STAR trial consists of a 2-sequence stepped wedge cluster-randomized trial, with 7 of the 14 county school districts randomly assigned to each sequence. A stepped wedge design with county-level cluster randomization was chosen based on feedback from community partners during initial conception of the study to ensure that all participating counties will eventually receive the STAR model. Furthermore, the stepped wedge design allows for sequential implementation of the screening and follow-up components of the STAR model, facilitating gradual integration of new technology and procedures into existing school and health systems. Consistent with this design, in each sequence, enhanced hearing screening is being rolled out first, followed by the addition of specialty telehealth follow-up. As shown in Figure 6, the key difference between the 2 sequences is that rollout of each of the 2 components is 1 year later in Sequence 2 than in Sequence 1. Consequently, there are 2 nested designs within the overall stepped wedge trial: a complete stepped wedge design to evaluate the screening component and an incomplete stepped wedge design to evaluate the specialty telehealth follow-up component (Figure 6).

Publicly available screening data from the Kentucky Department of Education suggest that current (usual) screening procedures vary significantly by county, which can result in significant variation by county in referral rates [39]. The nested design will assess follow-up outcomes for each sequence only after enhanced hearing screening is implemented, ensuring that hearing screening practices are consistent and comparable between participating counties. For the screening component’s complete stepped wedge design, Year 2 served as the baseline data collection period for the outcome of proportion screened, with all counties providing usual hearing screening according to state regulations. In Year 3 of the study, elementary schools in the 7 counties randomized to Sequence 1 provided enhanced hearing screening to all kindergartners, while schools in the 7 counties randomized to Sequence 2 continued to provide usual hearing screening. In Years 4 and 5, counties in both sequences will provide enhanced hearing screening. For the specialty telehealth follow-up component’s incomplete stepped wedge design, Year 3 serves as baseline for counties in Sequence 1, and Year 4 will serve as baseline for counties in Sequence 2, with elementary schools providing enhanced hearing screening and the usual follow-up process during each of these periods. Counties in Sequence 1 will provide specialty telehealth follow-up in Years 4 and 5, while counties in Sequence 2 will provide specialty telehealth follow-up in Year 5. The detailed trial protocol and the statistical analysis plan will be made available upon request and will be included as supplemental documents when publishing trial findings.

### Ethical Considerations

#### Human Subject Ethics Review

The study was approved by the University of Kentucky Institutional Review Board (IRB# 71393), with the University of Arkansas for Medical Sciences and Duke University IRBs serving as relying IRBs.

#### Adaptation and Implementation of Informed Consent

Informed consent procedures and study enrollment for Aim 1 (adaptation) were conducted by trained research personnel preceding the interview. A member of the study team reviewed the consent form with the participant and answered any questions. A copy of the consent was provided to the participant. The same procedures are being used for Aim 3 (implementation evaluation).

#### Trial Informed Consent

Per standard practice with school screening programs in public schools, children entering elementary school in participating schools receive the state-mandated hearing screen unless parents opt out. For the specialty telehealth follow-up, parental consent will be obtained in the form of a consent-to-treat document implemented in partnership with the OCSHCN clinic performing the telehealth follow-up. The informed consent was developed and adapted based on the feedback from Aim 1. The consent form for the specialty telehealth follow-up will be added to school enrollment paperwork. Child assent for the hearing screening will be conducted per usual procedures at the school, which involves stopping screening if a child shows unwillingness to participate. Similarly, should a child show unwillingness to participate during the specialty telehealth follow-up (eg, pushing the probe away, crying), the intervention will not be completed.

#### Privacy and Confidentiality

Primary outcome data for this trial will be obtained at the state level. The Kentucky Department Education, Cabinet for Health and Family Services, and Department of Medicaid Services will provide data to an honest broker. The honest broker will link children across data sets and de-identify the data before sharing with the research team for analysis. All data for the trial will be stored on secure servers with access limited to the study team. For Aims 1 and 3, all identifiable data are stored locally on secure servers at the University of Kentucky, and de-identified transcripts are being used for analysis.

#### Compensation Details

For Aim 1, participants received US $25 for participating in an interview or focus group. For Aim 3, participants receive US $25 for completing an interview or focus group each year of the trial that they participate. Children enrolled in the trial do not receive compensation, as they are participating in state-mandated hearing screening or school-based specialty telehealth follow-up with parental consent.

### Preliminary Adaptation

The core components of the STAR model include enhanced hearing screening; specialty telehealth follow-up; and streamlined communication between school staff, health care providers, and parents and caregivers. To tailor the STAR model to the Appalachian context, engagement with key stakeholders, including parents, educators, health care providers, and the CAB, guided adaptation of STAR using the ADAPT-ITT framework [36] during Years 1 and 2. Stakeholders were engaged in semistructured interviews addressing perceptions related to current school hearing screening, the referral process, and health care experiences with follow-up in the community (Multimedia Appendix 1). Stakeholders reviewed the core components of the STAR model and provided preferences regarding content, delivery, and potential challenges with each component. Rapid analysis of the resulting qualitative data directly informed adaptations [40,41]. Various aspects of the STAR model and its adaptations were presented to the CAB in the context of a theater test, with the goal of ensuring that the adapted STAR model is acceptable for the target population [36].

### Trial Participant Recruitment

All children enrolled in kindergarten at schools in the participating 14 counties are eligible to participate regardless of age, sex, race, or ethnicity (approximately 3600 children/year for a total of 14,400 children over 4 years). Inclusion in the trial is limited to school-aged children because the interventions being tested are specifically intended to improve hearing screening and access to care in rural schools. For children enrolled in kindergarten in a participating school, there are no additional exclusion criteria, such as age restriction, for study participation. We therefore anticipate enrolling children as young as 4 years and as old as 7 years. Children of families who opt out of screening will not be included in screening, per standard practice in participating schools. Children of families who decline to participate in the specialty telehealth follow-up will receive the usual follow-up.

In 2021-2022 (Year 1), all schools from the 14 participating counties were invited to participate in the trial in close collaboration with the Kentucky Commissioner of Education and the SAB. The commissioner worked with superintendents from the 14 participating counties (approximately 63 total schools), many of whom are also serving on the SAB, to finalize participation in the trial. The CAB guided integration of study information into consent documents that parents sign annually for school-based health programs, including hearing screening. Methods for parental communication were developed during the adaptation and theater testing in Aim 1.

Retention and attrition are not concerns in this trial because the design only requires participation once during annual kindergarten hearing screening. The trial will span Years 2-5 (2022-2026), and because of the natural advancement of children each academic year, new kindergartners participate each year of the trial, thus minimizing concerns for retention. Furthermore, data for the trial will be obtained at the population level from health and education entities and not from individual participants.

### Adaptation and Implementation Aims Recruitment

Participants in Aim 1 and Aim 3 include educational staff, parents, and key stakeholders from the 14 participating rural eastern Kentucky counties. Recruitment for Aim 1 occurred through a purposive sampling method, with the dual goal of reaching saturation and achieving diverse representation within each group. Similar recruitment is being used for Aim 3. Informed consent procedures and study enrollment are conducted by trained research staff. Participants fall into 3 major categories: (1) education stakeholders, (2) health care stakeholders, and (3) parents of early elementary-aged children and other key stakeholders.

Education stakeholders, including elementary school teachers, school nurses, administrative staff members, and speech-language pathologists involved in school hearing screening, were recruited using purposive sampling procedures for Aim 1. We aimed to achieve representation of several different types of staff, different counties, and different school sizes. For Aim 3, we are recruiting staff who are directly involved in delivering the STAR model in schools.

Health care stakeholders, including audiologists, otolaryngologists, and OCSHCN staff, were recruited using our professional networks of CAB members for Aim 1. We accessed perspectives from several groups of providers who work directly with children and their families. For Aim 3, we will prioritize health care providers who are directly involved in delivering specialty telehealth follow-up.

Parents of early elementary-aged children and other key stakeholders were recruited using purposive sampling procedures for Aim 1. Potential participants were identified by participating county educators, school board members, our OCSHCN co-investigator, CAB members, or other parent participants. For Aim 3, we are focusing on recruiting parents of children who participate in the STAR model.

### Randomization and Masking

County school districts (n=14) were randomized to 1 of 2 sequences using constrained randomization, which was used to balance important baseline covariates between sequences to increase statistical power and improve internal validity of the trial [42]. This involved calculating a balance score for each allocation and incorporating into the balance metric candidate covariates expected to be correlated with the primary outcomes (chosen a priori by study team consensus), including kindergarten enrollment per county (from 2021-2022 school year), number of kindergarteners screened per county (2021-2022 school year), and nearest OCSHCN clinic (as the stratification variable). Because the number of schools can vary widely by district, any allocations where either sequence included more than 36 schools (32 per sequence would be exactly balanced) were excluded. Of the remaining allocation schemes, the 5% with the best (ie, lowest) balance scores were kept. In the case where balance scores were tied at the 5% cutoff, ties were also included in the set of possible allocations. Independent statisticians performed the final randomization, and study statisticians will remain blinded to allocation until the primary analysis is complete. The allocation cannot be masked for other study team members, school staff, or parents for logistical reasons, but allocation is only revealed when required.

### Interventions

#### Intervention Condition: Enhanced Hearing Screening

Each kindergartener receives enhanced hearing screening once during the normal school screening period. The enhanced screening involves otoacoustic emissions (OAE) and tympanometry. This screening regimen was selected based on systematic reviews, practice guidelines for school screening, and results from our previous northwest Alaska trial [43-45]. Among various hearing screening protocols tested in our Alaska trial, regimens that included tympanometry were the most accurate. In particular, screening protocols incorporating tympanometry are the most sensitive in the detection of infection-related hearing loss, which is often observed in rural environments [44]. For children ages 3 years to 6 years, OAE and tympanometry performed better than pure-tone screening and tympanometry due to the challenges of getting young children to follow directions with participation-based audiometry tests [44]. Given that kindergarteners are the focus of the Appalachian STAR trial, we therefore selected OAE over pure-tone screening for this screening protocol.

OAE screening is conducted at 2, 3, 4, and 5 kHz frequencies, with 3 of 4 frequency pass criteria [46]. Criteria for referral include failure at 2 or more frequencies, with the test producing a pass or refer result. For tympanometry screening, criteria for referral include generation of a type B (flat) tympanogram or negative pressure less than –200 decapascal (daPa) per standard clinical guidance [47]. Results for tympanometry screening are displayed as a pass or refer result. A child is considered a referral if they do not pass OAE or tympanometry screening for one or both ears.

A hearing screening kit custom-designed for laypersons, such as teachers and school nurses who do not have specialized training in hearing, is provided to each school to implement the enhanced hearing screening, with districts retaining the equipment after conclusion of the trial. The STAR model screening kit includes all the tools necessary to complete the enhanced hearing screening, including tympanometry and OAE. Districts determine the appropriate staff members to conduct the enhanced hearing screening. This staff includes school nurses, speech-language pathologists, and/or teachers. Identified staff at each district receive training on the use of the hearing screening kit as part of the adapted STAR model procedures. Technology support and training are offered throughout the school year to support screening efforts. Because the screening equipment provides a pass or refer result, no clinical judgement is required for interpretation.

#### Control Condition: Usual Hearing Screening

Schools in the control condition continue usual hearing screening, with protocols varying by school. During Aim 1, we determined the screening protocols, devices, and practices used across participating schools.

#### Intervention Condition: Specialty Telehealth Follow-Up

Details of the specialty telehealth follow-up intervention were determined based on stakeholder feedback in Aim 1 and input from the CAB. Follow-up will be conducted only for children who refer (eg, fail) either OAE or tympanometry screening. Specialty telehealth follow-up will be conducted by school staff according to each school district’s preferred assignment of responsibilities; these individuals may include teachers, speech-language pathologists, or school nurses. Although details of the specialty telehealth follow-up will be determined during the adaptation phase of the STAR model, elements will include a lay-friendly platform that connects to tools, such as an otoscope for taking images of the ears; tympanometry; additional hearing testing; and possibly vital signs such as blood pressure and temperature. If a child receives a referral from hearing screening, school staff will complete the established procedure for the specialty telehealth follow-up with an audiologist. This will consist of asynchronous transfer of screening information (such as images of the ears) from school staff to an audiologist affiliated with a state-run OCSHCN clinic. The audiologist will provide a clinical assessment and recommendations for management to the school, family, and primary care provider. Results of the audiologist consult will be provided back to the school using the STAR model technology platform. Families will also receive results through email or SMS text messaging, from which a report can be viewed, printed, and shared if desired.

#### Control Condition: Usual Follow-Up

The control condition for specialty telehealth follow-up will be the usual process followed in Kentucky schools, which typically consists of a letter notifying parents that their child received a referral from screening and will need follow-up. Year 3 is the comparator for Sequence 1 counties, and Year 4 will be the comparator for Sequence 2 counties. As is currently standard across Kentucky and nationally, parents will be responsible for arranging follow-up for their child. State-run OCSHCN clinics see children who receive referrals from school screening for follow-up free of charge as standard practice, and this resource will continue to be available to families within the usual care sequence.

### Implementation Approach

#### Overview

Certain components of the implementation process were developed as part of Aim 1. Based on stakeholder feedback through qualitative interviews and CAB and SAB meetings, we adapted additional features of the STAR model to facilitate integration of enhanced hearing screening and specialty telehealth follow-up within the rural Appalachian school system. Major components of the implementation strategy designed a priori to Aim 1 stakeholder feedback are described in the following sections.

#### Screening Equipment

The overall intent of the STAR model is to deliver an all-in-one solution for school hearing screening. All equipment, accessories, consumable supplies, and supplementary materials are delivered in a single case. As core components of the intervention, an OAE device and a handheld tympanometer are included in the equipment. For the purpose of conducting the specialty telehealth follow-up intervention, screening kits will additionally include an otoscope. Otoscopes will be used by school screeners for children who are referred for screening to obtain images of the ears. These images will be asynchronously reviewed remotely by an OCSHCN audiologist in conjunction with screening results. We collected stakeholder feedback during Aim 1 to select the best existing software and hardware solutions and customized a novel platform to meet the needs of local partners.

#### Training and Technical Assistance

STAR enhanced hearing screening is intended to be delivered by existing school staff who may or may not have previously been engaged in other forms of hearing screening. These screeners may include school nurses, speech-language pathologists, and teachers who have varying degrees of clinical knowledge and skills. As such, the training is designed to be lay-friendly and tailorable to the needs of each county’s screener population.

Training sessions occur at each district, last 1 hour to 2 hours, and are delivered either in person or virtually. Content for education staff includes the use of the technology and performance of the screening tools. Content for clinical partners will cover the technology platform for specialty telehealth follow-up, which will include asynchronous review of hearing screening results and otoscopy images. Training is designed to be tailorable to the preferences of each county, with a variety of usable models (eg, direct training of all screeners, train-the-trainer approach). Per community preferences, the team developed materials that can be referenced and reviewed outside of training sessions at screeners’ convenience. Screeners receive a certificate attesting to their completion of training for the STAR enhanced hearing screening protocol. The study staff maintain a log of training attendees and verification of screeners as part of the fidelity measures.

The study team supports a dedicated phone line and email address for communication with school and clinical partners. This line is staffed during regular business hours. The study team aids with coordination of study logistics and troubleshooting of equipment and software. However, given the implementation aims of this study to be a community-administered intervention, the study team refrains from directly administering hearing screening or providing clinical review of data.

#### Implementation Adaptation

As defined within Aim 3 and guided by ADAPT-ITT, data-driven process adaptation occurs at predetermined time points after each school year (Figure 5). At these time points, the study team analyzes feedback from qualitative data and from CAB discussions to identify potential modifications to the STAR model. Core components of the STAR model remain unchanged to ensure adequate measurement of the effectiveness of the intervention. Enhanced hearing screening for kindergarteners will always involve OAE screening and tympanometry, and there will always be a pathway for specialty telehealth follow-up. However, certain elements of the intervention and its implementation approach are subject to change, based on qualitative feedback. Potential adaptations may include changes to training activities, the consent process, time and location of screening, screening personnel, screening hardware, school and clinical software interfaces, and communication pathways. Adaptations to STAR intervention technologies will be deployed uniformly across sequences and schools to ensure consistency in the enhanced hearing screening process. However, adaptations to screening personnel, training activities, and time and location of screening are tailorable according to individual county preference and may vary within or between sequences. This is to ensure that the STAR intervention fits within the existing infrastructure of each rural county and is deployed in line with community preferences.

Both formal qualitative data from interviews and informal discussions from CAB meetings are being used to gather feedback on proposed changes during implementation adaptation. We will query whether modifications improved the desired implementation outcomes and successfully addressed issues raised in the prior year to determine whether these modifications are maintained, modified further, or discarded between years. Through this process of iterative feedback and revision, periodic implementation adaptation is anticipated to not only improve the STAR implementation process with each iteration but also expand the range of tailorable variations to fit community needs. This will reinforce the overall flexibility and scalability of the STAR model within the implementation aim of this trial.

### Primary Outcomes

The trial has 2 primary outcomes: (1) proportion of kindergarteners screened for hearing loss and (2) proportion of referred kindergarteners (ie, those who did not pass screening) who receive specialty follow-up within 60 days of screening date

Prior to the rollout of enhanced screening, we collected baseline data during Year 2 (2022-2023) from education and health care agencies on the primary outcomes, including proportion of children screened for hearing and the proportion of referred children who received specialty follow-up. Proportion of children screened will be measured through school-based reporting mechanisms already used to monitor school-based health programs across the state. Schools will keep a record of children referred from screening per current practice in each school.

An electronic health record query of ear and hearing visits at state-run OCSHCN clinics, which are the primary source of specialty ear and hearing care for counties participating in the trial, will be used to indicate successful initiation of specialty services. We will also obtain Medicaid claims data on ear and hearing encounters for those who may seek services outside of OCSHCN clinics. Based on our previous trial, collecting electronic health record data for 60 days from screening date is sufficient to determine differences in rates of follow-up [37,48].

### Implementation Outcomes

To successfully implement the STAR model and prepare for scale-up beyond the participating counties, it is essential to understand the dynamic factors within schools, communities, and health care settings that influence implementation outcomes of the STAR model. Understanding these factors is essential to sustain STAR within rural Kentucky schools, as well as to develop implementation strategies to sustain and scale the STAR model beyond rural Kentucky.

For this trial, we are using the taxonomy of implementation outcomes by Proctor et al [49] to assess acceptability, appropriateness, feasibility, reach, and fidelity of the STAR model at the school level, as defined in Table 1. A multilevel, mixed methods assessment is being used to assess implementation factors and outcomes. Quantitative data sources include trial process records, surveys, and existing county- or school-level artifacts (eg, communication documentation with parents). Qualitative data sources include interviews or focus groups with a subset of the stakeholders, including educational staff, health care providers, and parents. The number of participants planned for interviews is based on predetermined targets regarding numbers needed to attain saturation and representation of key perspectives. Interviews occur annually beginning in 2023-2024 to understand implementation factors influencing each component of the STAR model as it is rolled out in phases in participating counties. Stakeholders are engaged in quantitative and qualitative data collection at multiple time points that are applicable within trial operations. Data collection will occur after enhanced hearing screening is completed in schools for educational staff and after specialty telehealth follow-up has concluded for health care staff and parents.

**Table 1:** Implementation outcome measures for Aim 3 of the Appalachian Specialty Telemedicine Access for Referrals (STAR) trial.

Implementation outcome	Definition	Data source	Data collection timing^a^
Appropriateness, acceptability, feasibility	STAR model is appropriate, acceptable, and feasible for participating schools	Brief questionnaires (ORIC^b^, IAM^c^, AIM^d^, FIM^e^) [45,46] for all implementation actors at schools	For questionnaires on enhanced screening: Year 3 for Sequence 1 and Year 4 for Sequence 2; for questionnaires on specialty telehealth follow-up: Year 4 for Sequence 1 and Year 5 for Sequence 2
Reach	Representativeness of children reached by the STAR model based on demographic factors such as race/ethnicity and county	STAR process records, school data	Annually during Years 3-5
Fidelity	Extent to which core components of the STAR model are implemented as intended, contextualized using the CFIR^f^ framework of factors influencing fidelity [41].	Interviews with education and health care stakeholders, parents	Annually during Years 3-5
Fidelity	Degree of adherence to core components; adherence thresholds will be determined as core components are finalized through theatre testing	Fidelity checklist filled out by school or study staff	Annually during Years 3-5

^a^Year 3 is the 2023-2024 academic year; Year 4 is the 2024-2025 academic year; Year 5 is the 2025-2026 academic year.

^b^ORIC: Organizational Readiness for Implementing Change.

^c^IAM: Intervention Appropriateness Measure.

^d^AIM: Acceptability of Intervention Measure.

^e^FIM: Feasibility of Intervention Measure.

^f^CFIR: Consolidated Framework for Implementation Research.

#### Appropriateness, Acceptability, and Feasibility

To assess appropriateness, acceptability, and feasibility, key informants complete modified versions of the following brief, validated questionnaires: Intervention Appropriateness Measure, Acceptability of Intervention Measure, Feasibility of Intervention Measure, and Organizational Readiness for Implementing Change [50,51]. Surveys are completed annually to assess outcomes across various STAR components as they are rolled out. We will bolster our understanding of appropriateness, acceptability, and feasibility through qualitative feedback from key informants. Probes have been developed to yield perspectives on the fit of the STAR model within school contexts, competing priorities, and ease of use.

#### Reach

We are evaluating the representativeness of children reached by the STAR model based on demographic factors such as race/ethnicity and county. This will provide valuable context for the primary outcomes, which measures overall proportions of children reached by the screening and specialty telehealth follow-up components of the STAR model.

#### Fidelity

We are evaluating fidelity by assessing adherence to the intervention, as well as the context for how the intervention is implemented [49]. We are using several sources of pragmatic real-world data. To reduce implementer burden, fidelity is primarily assessed by the research team using a structured fidelity checklist of the intervention’s core components. In addition to the use of checklists, fidelity to the specialty telehealth follow-up component will also be measured by assessing time to specialty follow-up through comparison of the date of specialty ear and hearing encounter to the date of screening. This measure will provide additional context to the second primary outcome by measuring the average time to follow-up as a numeric variable rather than as a categorical variable (ie, follow-up within 60 days or not).

Last, fidelity is assessed using qualitative data collection during semistructured interviews. For educational staff, probes have been developed to ask about how various facets of the STAR intervention were used (ie, which grade levels were screened, what screening tests were used, referral criteria, and parental communication method). Similarly, for health care staff, probes may ask about whether follow-up care was completed using the telehealth interface developed for the STAR intervention. On an annual basis, quantitative and qualitative data will be compared to develop reports on fidelity at the individual school level and to support adaptations.

#### Implementation Factors

This effectiveness-implementation trial includes adaptation phases both before and between the key years of the STAR intervention deployment (ie, Years 3-5). The adaptation process uses targeted modifications to the STAR implementation approach derived from qualitative feedback from key stakeholders. First, we will seek to develop an explanatory model for the aforementioned implementation outcomes by analyzing various implementation factors. The primary data source for this component of the implementation evaluation will include the semistructured interviews and focus groups conducted with key informants during each school year. Using the Consolidated Framework for Implementation Research (CFIR) [41], we will operationalize multiple factors that influence implementation outcomes in the school setting in 4 domains: components of the STAR model (Intervention), how it is implemented (Process), the school environment for implementation and integration with the health care setting (Inner Setting), and the external policies and people facilitating implementation (Outer Setting; Table 1). These implementation factors will be analyzed with respect to each component of the STAR model to inform adaptations and scalability [40].

### Statistical Analysis

#### Sample Size

To calculate power for a cross-sectional stepped wedge design with a binary outcome, we used the approach from Hemming et al [52,53] based on a comparison of 2 proportions, incorporating design effects to account for clustering by county school district, repeated measures on the same cluster, and *t*-based inference to accommodate the small number of clusters (ie, 14 counties). The design is cross-sectional rather than cohort because data are collected from different children each year of the study. Assumptions for power calculations for the primary outcomes are summarized in Table 2.

**Table 2:** Power calculation assumptions for primary outcomes of the Appalachian Specialty Telemedicine Access for Referrals (STAR) trial.

Parameter		Enhanced hearing screening	Telehealth specialty follow-up
**Outcomes and intervention effect**			
	Outcome	Screening	Specialty care within 60 days of referral
	Kindergarteners included in analysis	All	Those screened and referred
	Proportion in control periods, %	36^a^	20
	Proportion in intervention periods, %	56	60
	Intervention effect, percentage point	+20	+40
**Sample size assumptions**			
	Total kindergarteners per year, n	3635^b^	101^c^
	Clusters (counties) per sequence, n	7	6^d^
	Median cluster size (m)	201	6
	Cluster size CV^e^	0.73	0.91
**Correlation structure assumptions^f^**			
	Within-period ICC^e^	0.10	0.10
	Correlation structure over time	2-period decay	2-period decay
	Cluster autocorrelation (CAC)	0.8	0.8
	Calculated power^f^, %	>90	90

^a^Based on population weighted average of 2018-2019 reported county level screening rates.

^b^Based on 2019-2020 county level kindergarten enrollment in 14 counties.

^c^Based on assumption that each of the 2018-2019 reported county level screening rates improved 20 percentage points and the referral rate was approximately 5% in all counties.

^d^Based on previous data, 1 county expected to have 0 referrals. The power calculation was thus conservatively based on one county being excluded from each of the 2 trial sequences.

^e^CV: coefficient of variation.

^f^Conservative assumptions estimated based on the method by Hayes and Moulten [54] to estimate the within-period intracluster correlation coefficient (ICC) using published data at the county level.

^e^ICC: intracluster correlation coefficient.

^f^Based on comparison of 2 proportions within a stepped-wedge design [55,56].

#### Screening Outcome

Power for the screening outcome was calculated based on kindergarten enrollment from the 2019-2020 school year (n=3635) and screening rates reported from the 2018-2019 school year due to lags in screening data availability and concern for the potential impact of COVID-19 on school-based preventive health screenings. Data from all 14 participating county school districts were used, with an expected median annual county kindergarten enrollment (ie, cluster-period size) of 200, a baseline screening rate of 36%, and a coefficient of variation (CV) of cluster size of 0.73.

We estimated a within-period intracluster correlation coefficient (ICC) using a formula based on the CV of the outcome. The CV was estimated using a published strategy [54]: County-specific proportions of the screening outcome were assumed to be normally distributed, centered on the control arm proportion with a between-cluster standard deviation derived from an assumed range for 95% of the county-specific proportions (ie, for a width of ∼4σ_B_). Given the wide range of 2018-2019 county-specific screening rates reported, we estimated a likely half-width of 30% for the interval around the baseline screening rate of 36% (π). Using the published strategy based on the following formula, ICC=(σ_B_^2^)⁄[π(1-π)] yielded a corresponding ICC of ~0.10. We conservatively assumed a cluster autocorrelation of 0.8 and a 2-period decay structure between measurements within the same cluster over subsequent time periods. We assumed a 2-period decay structure in the design phase rather than an alternate less realistic structure such as exchangeability to protect against an underpowered trial [55,56].

Given a 2-sided α of .05, with the aforementioned cluster-period size and clustering assumptions and assuming a complete stepped wedge design with 2 sequences of 7 clusters (counties) each (Figure 6), the study will have greater than 90% power to detect an increase in screening of 20 percentage points (36% vs 56%) using a 2-sided test of difference in proportions [52,56,57].

#### Follow-Up Outcome

**Figure 7:**
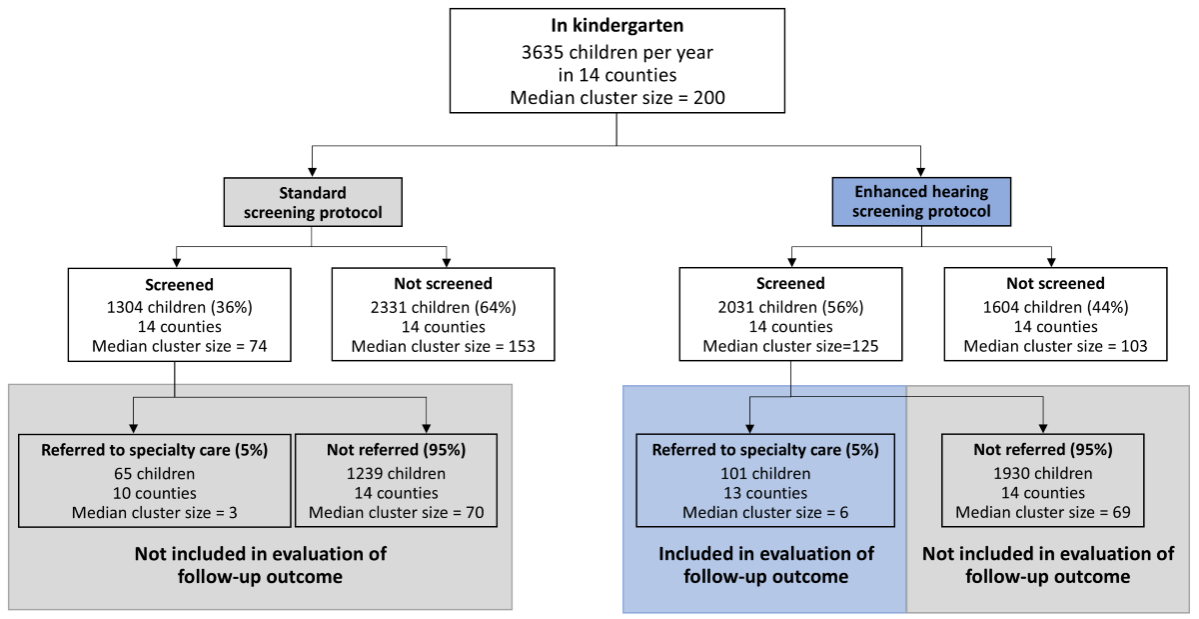
Estimating cluster size for specialty telehealth follow-up outcome based on prior school data.

To calculate power for the follow-up outcome, it was necessary to estimate the number of students who will be referred for follow-up each year in each county. To do this, we multiplied the assumed county-level screening proportion under enhanced hearing screening (56%) by the expected county-level kindergarten enrollment and by the expected referral rate of 5% (a conservative assumption given the lack of current referral data in Kentucky). It is possible that, even after the introduction of enhanced screening, one of the smaller counties may have zero referrals. With this assumption, we expect a median cluster-period size of 6 referred children (ie, per county per year), a CV of cluster size of 0.91, and 6 counties per sequence (Figure 7).

To be conservative, we assumed the follow-up outcome proportion to be 20% for children in the control condition (ie, enhanced hearing screening only). With similar assumptions about ICC (0.10) and cluster autocorrelation (0.80) as for the screening outcome, a 2-sided α of .05 and assuming an incomplete stepped wedge design with 2 sequences of 6 clusters each (Figure 6), the study will have 90% power to detect an increase of at least 40 percentage points in the proportion of referrals resulting in specialty care (ie, 20% vs 60%).

#### Analysis of the Primary Effectiveness Outcomes

For analysis of the first primary outcome (proportion of children screened for hearing loss), the intention-to-treat population will be all students enrolled in kindergarten at the elementary schools across 14 counties of eastern Kentucky participating in the study, at the time of their school’s screening for hearing loss, over Years 2-5. For analysis of the second primary outcome (proportion of referrals resulting in specialty follow-up within 60 days of the date of screening), the intention-to-treat population will be all kindergarten students referred for follow-up during their school’s enhanced hearing screening period (Years 3-5 for the first sequence, Years 4-5 for the second sequence), regardless of whether their parent or guardian opts in to the specialty telehealth follow-up intervention.

Child-level outcomes will be analyzed using generalized estimating equations (GEEs) in order to accommodate correlation within counties and schools that is expected to arise due to the repeated cross-sectional data obtained over time. Moreover, the GEE approach will provide population-averaged intervention effects that are relevant to public health implementation [58,59]. More specifically, binary outcomes at the child level will be analyzed assuming a binomial outcome distribution and will include the following terms: an indicator for whether the county (ie, cluster) is observed under the usual care or intervention condition during the cluster-period; time effects to account for the potential confounding effect of time and fixed effects for the variables used in the constrained randomization procedure (kindergarten enrollment, baseline screening rate, and which OCSHCN clinic serves the county); and prespecified adjustment for sociodemographic characteristics (eg, gender, race, ethnicity, and free-reduced lunch eligible) of children to account for potential confounding.

Results will be reported on the relative scale as risk ratios, and absolute effects will be reported as risk differences, both with 95% confidence intervals [60]. To do this, the identity- and log link will be used to estimate absolute and relative effects, respectively. In the event of issues with convergence of either the log-binomial or identity-binomial GEE models, we will use the modified Poisson approach [61] with log or identity link, as needed. If further convergence issues arise with the log link, we will instead use the standard logit-binomial GEE and report odds ratios as the estimate of a relative effect, and if further convergence issues arise with the identity link, the Gaussian distribution will be used with identity link to estimate absolute effects [62]. Standard errors will be estimated using the robust sandwich variance estimator using appropriate small-sample corrections to obtain valid standard errors [63]. The GEE approach assumes that data are missing completely at random, thus this assumption will be checked.

Within-period ICCs will be calculated and reported based on baseline outcome levels across all counties. Given that the overall intervention (enhanced school hearing screening plus specialty telehealth follow-up) will be considered effective if both tests are significant, a 5% significance level will be used for each of the 2 primary outcomes with no alpha penalty [64].

#### Missing Data

Missing data for the screening outcome will likely be in the form of loss of reporting from some schools or from closing or merging of schools. Detection of missing data at the child level will not be possible, as children enrolled but not present will count as not being screened. Missing data for the specialty follow-up outcome may occur if referred children move out of the region or receive follow-up care from providers outside our data observation scope. Child-level enrollment date ranges will help us determine if the former has happened, but we cannot track the latter. Therefore, our analysis will assume that, if a specialty follow-up visit is not recorded in our data (and the child remains enrolled in a regional school), the visit did not take place. Depending on the pattern of missingness at the school level, we may use an inverse probability weighting or multiple imputation approach. We will use complete case analysis only if data loss is thought to be missing completely at random.

### Qualitative Analysis

Interviews and focus groups are recorded, transcribed, and analyzed manually or using Atlas.ti qualitative software depending on the volume of data collected at each analysis phase. Administrative data, including CAB meeting notes and communication logs between sites and study staff, are also included in content analyses. Coders use an iterative process to develop a framework, categories, and coding plan for each phase of analysis. Interview and focus group transcripts are coded with respect to established CFIR domains [40,41]. To ensure intercoder reliability, discrepancies are resolved through discussion among the qualitative analysis team. The study co-investigators review the content generated from analysis.

A multiphase mixed methods design is being used to evaluate program implementation and targeted adaptation. Quantitative and qualitative data will be combined to inform predetermined implementation outcomes of appropriateness, acceptability, feasibility, reach, and fidelity. Rapid content analysis of qualitative data will be integrated with descriptive analysis of quantitative data consistent with a mixed methods design.

### Monitoring and Risk Assessment

The STAR model poses minimal risk for participating children and introduces no more than ordinary risk encountered during a routine ear and hearing exam. Since this is a minimal risk study, a Data Safety Monitoring Board is not required; however, a Data Safety Monitoring Plan is in place.

### Data Management and Security

Data and consents from the qualitative aspects of the study are stored in a secure Research Electronic Data Capture (REDCap) database locally at the University of Kentucky. Study personnel obtain consent and manage the coding system linking participant names to identification numbers, which is maintained in an encrypted, password-protected database separate from the data. Trial data are obtained at the population level, with a limited data set that links school hearing screening results and ear and hearing encounters. All identifying data for the trial are removed, and unique identifiers are assigned prior to receipt by the study team. There are no key linking identifiers to trial data. The database design incorporates all necessary logic and range checks and prompts. The data manager is responsible for ensuring the accuracy, completeness, legibility, and timeliness of the data reported. The University of Arkansas for Medical Sciences serves as the data coordinating center, housing the secure research database and overseeing the data architecture, sharing, and management.

The multiple principal investigators (MPIs) verify that all study data generated, documented, and reported are compliant with the protocol and the applicable regulatory requirements for good clinical practices. Despite population-level data collection for the trial, interval quality assurance audits are conducted and overseen by the MPIs. Reports are developed at regular intervals to monitor data accuracy. Monitoring is conducted using web-based data validation rules and data manager review.

The MPIs are responsible for ensuring participant safety for the study. Although no adverse events are anticipated during data collection for the adaptation and sustainability evaluation of the STAR model, should an individual experience any sensitivities during an interview, the interviewers are trained for an appropriate response, including concluding the interview early. An iterative process has been put into place to adapt interview questions and study personnel training as needed to address this possible risk. In the case of an adverse event or unanticipated problem, such as breach of confidentiality, the MPIs will be notified within 24 hours. If required, the MPIs will report the adverse event or unanticipated problem to the appropriate IRB within 7 days and will determine necessary action, from changes to the protocol to cessation of activity, in compliance with IRB oversight.

### Dissemination Plan

This study complies with the National Institutes of Health (NIH) Data Sharing Policy, Policy on the Dissemination of NIH-Funded Clinical Trial Information, and the Clinical Trials Registration and Results Information Submission rules. The trial was registered at ClinicalTrials.gov, and results from the trial will be submitted to ClinicalTrials.gov. Adhering to the community-based nature of the proposal, results will be shared with Kentucky communities. Dissemination efforts will be made at the end of the trial to communicate study findings to members within each of the participating communities. We will also make our results available to the broader scientific community through presentations at regional and national scientific meetings, as well as through publications. The input and collaboration of others studying similar scientific questions in this discipline will be welcomed. All finalized data sets, protocols, and study materials will be made publicly available upon request with necessary approvals and execution of a formal data use agreement.

## Results

The stepped wedge trial began in September 2022 with baseline data collection. Adaptations of the STAR model were completed by summer 2023 based on data collected as part of Aim 1. Randomization was conducted in 2023, and the phased rollout of the STAR model began in the 2023-2024 academic year. The trial is expected to conclude in May 2026. Final data analysis is planned to begin in June 2026, and publication of results is expected in 2027.

## Discussion

### Appalachian STAR Trial

The Appalachian STAR trial represents a unique opportunity to demonstrate that school-based telehealth can improve access to specialty hearing care for rural children. Pediatric ear and hearing care constitutes a critical area for intervention, as the WHO estimates that up to 75% of childhood hearing loss is preventable in underserved communities, and the repercussions of untreated childhood hearing loss continue into adulthood [14,15,65-67]. Kentucky and greater Appalachia exemplify how rurality often predisposes to worse ear and hearing outcomes due to limited access to specialty care [16-18,20].

This trial places communities at the center, using formative work and implementation evaluations to adapt and implement a community-informed intervention designed to be sustainable and scalable. Community partnership in this trial spans active CAB involvement to engagement with school staff and leadership, health care providers, and parent partners. Strong partnerships at the state level and support for this study from Kentucky state leadership and stakeholders have set the stage for sustainability and scalability of the STAR model. The study also leverages existing infrastructure and programs in state-run clinics, facilitating follow-up and removing barriers to care through creation of a model that can be replicated for other preventive screenings within the state.

The Appalachian STAR trial is designed to fill gaps in the evidence to influence school screening policy and evaluate a novel intervention with significant potential to broadly change access to specialty care for rural children. There is currently no national standard for school hearing screening, with different states and even different counties within a single state variably implementing screenings with a range of protocols. This trial evaluates the effectiveness and implementation of an evidence-based enhanced hearing screening protocol that incorporates tympanometry, a middle ear assessment we have previously shown significantly improves the accuracy of screening in rural Alaskan communities where infection-related hearing loss is common [44]. The Appalachian STAR trial will be the first to evaluate the effectiveness of this screening protocol when conducted by school-based screeners in an environment that is prototypical of other rural contexts in the United States. As a result, evidence from this trial may inform policy change and implementation of a standardized, evidence-based school screening protocol statewide in Kentucky and across other rural states, placing Kentucky at the forefront of transforming preventive school hearing screenings throughout the United States. Our second primary outcome will establish the effectiveness of specialty telehealth follow-up in the school setting, while our supplemental implementation aims will inform the overall adaptability and scalability of the STAR model. It is critical to understand the various implementation factors that facilitate successful integration of this model of care delivery into rural schools and clinical contexts. Once proven successful with hearing, the STAR model could be applied to address multiple serious health disparities that begin in childhood and affect underserved rural populations for a lifetime, such as vision loss, behavioral health, development, and obesity [68].

### Limitations

A pragmatic stepped wedge trial design such as ours often imposes challenges to maintain internal validity while ensuring broad applicability of the intervention in a real-world setting. To enhance internal validity, we balanced important covariates between the sequences that are expected to be correlated with the primary outcome. Cluster-randomized trials have inherently lower power than parallel design trials; however, this design is important for testing an intervention that is intended to be implemented at the school level. Based on the anticipated sample size, we expect to have sufficient statistical power to detect effects of the primary outcomes. One limitation inherent in the study design is that it is not possible to mask participating schools and parents to allocation. A high degree of collaboration with community stakeholders is needed for integration of the STAR model in rural schools, and parents receive information about the program at the start of the academic year. However, the statistical team is masked to allocation until primary analysis is complete, and allocation is only revealed to members of the study team who need the information for managing the logistics of implementation. Another potential challenge is ensuring intervention fidelity across counties. Implementation of the STAR intervention involves screeners gaining familiarity with new technology and screening protocols, which could lead to inconsistent adoption. We have put several safeguards in place to avoid this, such as the development of training materials and incorporation of fidelity checklists. Additionally, there are 2 prespecified time points during the trial when community feedback from the Aim 3 implementation evaluations can be used to adapt the solution to address various implementation factors that may affect fidelity, improving overall success of integration and providing key insights on various modifiable components of the protocol to maximize uptake (Figure 5). These obstacles are not exclusive to this study and are common to other effectiveness-implementation trials.

### Conclusion

The Appalachian STAR trial is evaluating a new model of care that has great potential to improve access to specialty care for rural children. The evidence generated on school hearing screening may influence policy in Kentucky and other rural states, and, if effective, this novel model of specialty telehealth follow-up could be generalized to other preventable health disparities beyond hearing.

## Appendix

Multimedia Appendix 1 Aim 1 Interview Guides.

Multimedia Appendix 2 SPIRIT Checklist.

Multimedia Appendix 3 Peer review report by ZRG1 MOSS-*t* (50) - Center for Scientific Review Special Emphasis Panel RFA-RM-21-021: UNITE Transformative Research to Address Health Disparities and Advance Health Equity (U01) (National Institutes of Health, USA).

## Abbreviations

CAB: community advisory board
CFIR: Consolidated Framework for Implementation Research
CV: coefficient of variation
GEE: generalized estimating equation
ICC: intracluster correlation coefficient
IRB: institutional review board
MPI: multiple principal investigators
NIH: National Institutes of Health
OAE: otoacoustic emissions
OCSHCN: Office for Children with Special Health Care Needs
PCORI: Patient-Centered Outcomes Research Institute
REDCap: Research Electronic Data Capture
SAB: Stakeholder Advisory Board
STAR: Specialty Telemedicine Access for Referrals
WHO: World Health Organization

## Data Availability

All finalized data sets, protocols, and study materials will be made publicly available upon request with necessary approvals and execution of a formal data use agreement.
