# Supplementary material for: Improving Access to Specialty Care for Rural Children Using Enhanced Hearing Screening and Specialty Telehealth Follow-Up in Rural Kentucky Schools: Protocol for a Hybrid Effectiveness-Implementation Stepped Wedge, Cluster-Randomized Controlled Trial (Appalachian STAR Trial)
Source: JMIR Res Protoc. 2025 Aug 26;14:e77630. doi: 10.2196/77630 (PMC12421207; doi:10.2196/77630)
Supplement: Multimedia Appendix 1 [file resprot_v14i1e77630_app1.pdf]

Appalachian STAR Trial

<u>Education Stakeholder Interview Questions</u>

INTERVIEWER-BACKGROUND, EXPECTATIONS & INTRODUCTIONS

<u>Narrative Intro:</u> *Loss to follow-up is a widespread problem within school hearing screenings: many children who are referred to see a hearing specialist for further screening do not receive this follow-up care. Living in a rural area is one challenge to receiving follow-up care but there are others. This gap is addressable.*

Previous research that we did in rural Alaska sought to improve the school hearing screening process and ensure that children got follow-up care if needed. We used a clinic-based telehealth system to send information to a hearing specialist for evaluation. This approach worked very well. In schools that did not have telehealth follow-up, only 32% got follow-up with a hearing specialist. But in schools that did have telehealth follow-up, 68% received follow-up with a hearing specialist. We hope to do something similar here in Kentucky, but bring the telehealth follow-up into the school where the children are, instead of in a medical care or clinic setting.

We are working with stakeholders like yourself to understand how this telehealth intervention could work in Kentucky. We want to learn from your expertise to ensure we are setting up this program for success.

Thanks for sharing a little bit about yourself in the initial survey! It looks like you've been a _________ for _______ years (pull from REDCap).

- Can you tell me about your current role? *(*ask this question if needed)*

EXPERIENCE WITH CHILDHOOD HEARING LOSS

[For participants who reported experience with students with hearing difficulty on REDCap survey]

You mentioned on the survey that you've had experience with a student or students with hearing difficulty.

- Can you describe your experience?
- Can you describe any challenges of meeting the needs of students with hearing difficulty? (*(Probe: issues with communication, teaching in classroom, coordination of necessary care)*

HEARING SCREENING AND FOLLOW-UP PROCESS

[For participants who reported screening experience on REDCap survey]

You reported that you have experience with hearing screenings in your district.

- Can you walk me through from start to finish the process of screening students' hearing in your district? *(Looking for input on who, when, where, how, why; Who screens, equipment used, how screening is done, how results are communicated and to who, as well as any policy or school requirements followed; also need specific details on parental permission of health screenings in schools)*

- What are some challenges you've experienced with current or past hearing screening and/or follow-up processes?

TRIAL-SPECIFIC QUESTIONS

Appalachian STAR Trial Model

Narrative Intro: *[Show or explain image of enlarged graphic.] There are 4 parts to the model we would like to adapt and implement for Kentucky. We are calling this the Appalachian STAR Trial. "STAR" stands for Specialty Telemedicine Access for Referrals***.** *[Explains each of the components]. We would like your feedback on each of these 4 areas.*

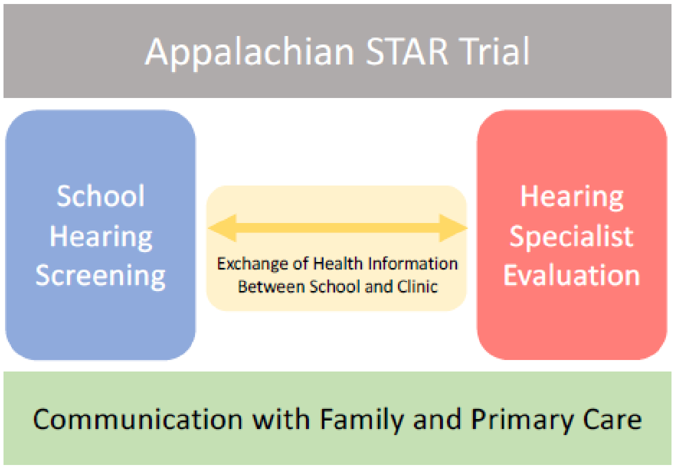

- Before we get started, are there other similar programs in your school/District? If so, how do they compare?

CORE COMPONENT: School Hearing Screening

- What are the most important challenges that we should anticipate and address within the school hearing screening process?
- Who are the key individuals to involve with the new screening process?

CORE COMPONENT: Hearing Specialist Evaluation

Narrative Intro: We want to look at how well the telehealth follow-up would work if performed in the school, maybe by school staff rather than a medical clinic off-site. This follow-up would be done shortly after the first hearing screening. It would include getting some additional information, such as images of the ears. This information would be sent to an audiologist or hearing healthcare specialist for review and care plan.

- What will be some challenges to implementing this model in your school/district?
- How can we ensure this model is feasible within current school infrastructure?

Narrative Intro: Our work in *rural Alaska showed students and families don't always get the information they need for follow up hearing care. At the same time, we noticed that hearing specialists had a hard time communicating directly with the school or teachers regarding a student's needs. We want to improve communication, so kids don't fall through the cracks. The next few questions ask you about improving this communication.*

CORE COMPONENT: Exchange of Health Information Between School & Specialty Clinic/Provider

- Generally, what kind of information exchange does your school have with clinics/medical offices in your community?
- How do the appropriate school personnel (e.g., SLP, teacher, special education) learn about whether or not students receive follow-up from a hearing clinic? (Prompt: interoffice memo, ROI between school/clinic, letter, phone call, etc.)
- When thinking about communication between the school and clinics/medical offices, what challenges might we encounter? How could we avoid communication breakdowns? *(probe: when, how, what should be shared; what should this exchange look like)*

CORE COMPONENT: Communication with Family and Primary Care

- Generally, what kind of information exchange does the school have with students' families and primary care providers?
- How does the school share health information with families? How about with primary care providers? *(Probes?___________)*
- When thinking about communication with families and primary care providers, what challenges might we encounter? How could we avoid communication breakdowns? *(probe: when, how, what should be shared; what should this exchange look like; previous work suggests text/email)*

OVERALL Appalachian STAR Trial Model

- In summary, when thinking about the four main parts of the Appalachian STAR model, what you said would work for your school/district right now was ______________? What you said might not work is ________________? Is that an accurate summary?
    - Is there anything else you would like to share that we haven't yet discussed?

- Who else should we talk with?

Appalachian STAR Trial

Healthcare Stakeholder Interview Questions (excludes school SLPs)

INTERVIEWER-BACKGROUND, EXPECTATIONS & INTRODUCTIONS

Narrative Intro: *Loss to follow-up is a widespread problem within school hearing screenings: many children who are referred to see a hearing specialist for further screening do not receive this follow-up care. Living in a rural area is one challenge to receiving follow-up care but there are others. This gap is addressable.*

Previous research that we did in rural Alaska sought to improve the school hearing screening process and ensure that children got follow-up care if needed. We used a clinic-based telehealth system to send information to a hearing specialist for evaluation. This approach worked very well. In schools that did not have telehealth follow-up, only 32% got follow-up with a hearing specialist. But in schools that did have telehealth follow-up, 68% received follow-up with a hearing specialist. We hope to do something similar here in Kentucky, but bring the telehealth follow-up into the school where the children are, instead of in a medical care or clinic setting.

We are working with stakeholders like yourself to understand how this telehealth intervention could work in Kentucky. We want to learn from your expertise to ensure we are setting up this program for success.

Thanks for sharing a little bit about yourself in the initial survey! It looks like you've been a _________ for _______ years (pull from REDCap).

- Can you tell me about your current role? *(*ask this question if needed)*

EXPERIENCE WITH CHILDHOOD HEARING LOSS

[For participants who reported experience with hearing difficulties in school-aged children on REDCap survey]

You mentioned on the survey that you've had experience with hearing difficulties in school-aged children.

- Can you describe your experience?

HEARING SCREENING AND FOLLOW-UP PROCESS

[For participants who reported experience with school hearing screening]

You reported that you have experience with hearing screenings in the school.

- Can you describe your experience?

[For participants who reported experience with school hearing screening follow-up on REDCap survey]

You reported that you have experience with school hearing screening follow-up in your clinical practice.

- Can you walk me through from start to finish the follow-up process for children who have been referred from their school hearing screenings? *(Looking for input on who, when, where, how, why. How results are communicated and to who, any policy or school/healthcare provider communication requirements followed, etc.)*
- What are some challenges you've experienced with current or past hearing screening follow-up processes?

TRIAL-SPECIFIC QUESTIONS

Appalachian STAR Trial Model

*<u>Narrative Intro:</u> [Show or explain image of enlarged graphic.] There are 4 parts to the model we would like to adapt and implement for Kentucky. We are calling this the Appalachian STAR Trial. "STAR" stands for Specialty Telemedicine Access for Referrals***.** *[Explains each of the components]. We would like your feedback on each of these 4 areas.*

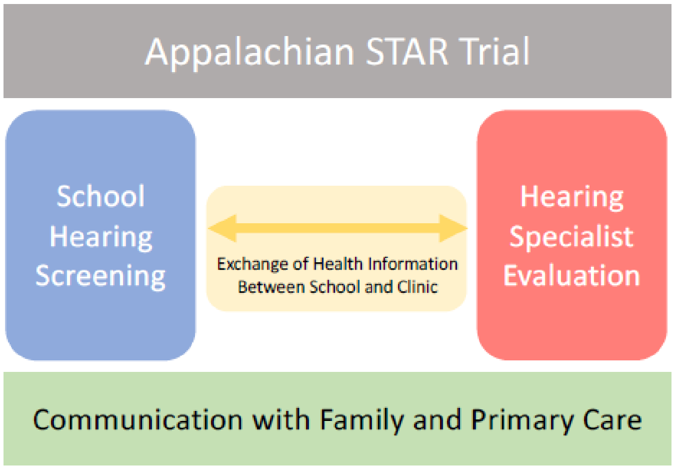

- Before we get started with the details of this model, are there other similar follow-up programs in your clinic/office? If so, how do they compare?

CORE COMPONENT: School Hearing Screening

- What are the most important challenges that we should anticipate and address within the new school hearing screening process?

CORE COMPONENT: Hearing Specialist Evaluation

Narrative Intro: *We want to look at how well the telehealth follow-up would work if performed in the school, maybe by school staff rather than a medical clinic off-site. This follow-up would be done shortly after the first hearing screening. It would include getting some additional information, such as images of the ears. This information would be sent to an audiologist or hearing healthcare specialist for review and care plan.*

[For participants who reported experience with telehealth on REDCap survey]

You mentioned on the survey that you've had experience with telehealth.

- Can you describe your experience?

[For all participants]

- How could we ensure that this model is feasible for hearing healthcare specialists? (*Probe: How might this model integrate into your everyday clinical schedule?*)

[For receiving audiologists who reported experience with school hearing screening follow-up OR experience with telehealth in REDCap responses]

- What are some potential challenges or barriers to receiving and managing telehealth consults from schools for children that referred school hearing screening?

CORE COMPONENT: Exchange of Health Information Between School & Specialty Clinic/Provider

Narrative Intro: *Our work in rural Alaska showed students and families don't always get the information they need for follow up hearing care. At the same time, we noticed that hearing specialists had a hard time communicating directly with the school or teachers regarding a student's needs. We want to improve communication, so kids don't fall through the cracks. The next few questions ask you about improving this communication.*

- Generally, what kind of information exchange does your clinic/office have with schools in the community? *(probe: school nurses, school administrators?)*
- How do the appropriate clinic personnel learn about whether or not students need follow-up after a school hearing screening? (Probe: parent/guardian contacts clinic, interoffice memo between school and clinic, ROI between school/clinic, letter, phone call, etc.)
- When thinking about communication between the school and your clinic/office, what challenges might we encounter? How could we avoid communication breakdowns? *(probe: when, how, what should be shared; what should this exchange look like)*

CORE COMPONENT: Communication with Family and Primary Care

- How does your clinic/office share health information with families? What works best?

- When thinking about communication with families and schools, what challenges might we encounter? How could we avoid communication breakdowns? *(probe: when, how, what should be shared; what should this exchange look like; previous work suggests text/email)*

OVERALL Appalachian STAR Trial Model

- In summary, when thinking about the four main parts of the Appalachian STAR model, what you said would work for your clinic/office right now was ______________? What you said might not work is ________________? Is that an accurate summary?
    - Is there anything else you would like to share that we haven't yet discussed?

- Who else should we talk with?

Appalachian STAR Trial

Healthcare Stakeholder Interview Questions: School SLPs & Nurses

INTERVIEWER-BACKGROUND, EXPECTATIONS & INTRODUCTIONS

Narrative Intro: *Loss to follow-up is a widespread problem within school hearing screenings: many children who are referred to see a hearing specialist for further screening do not receive this follow-up care. Living in a rural area is one challenge to receiving follow-up care but there are others. This gap is addressable.*

Previous research that we did in rural Alaska sought to improve the school hearing screening process and ensure that children got follow-up care if needed. We used a clinic-based telehealth system to send information to a hearing specialist for evaluation. This approach worked very well. In schools that did not have telehealth follow-up, only 32% got follow-up with a hearing specialist. But in schools that did have telehealth follow-up, 68% received follow-up with a hearing specialist. We hope to do something similar here in Kentucky, but bring the telehealth follow-up into the school where the children are, instead of in a medical care or clinic setting.

We are working with stakeholders like yourself to understand how this telehealth intervention could work in Kentucky. We want to learn from your expertise to ensure we are setting up this program for success.

Thanks for sharing a little bit about yourself in the initial survey! It looks like you've been a _________ for _______ years (pull from REDCap).

- Can you tell me about your current role? *(*ask this question if needed)*

EXPERIENCE WITH CHILDHOOD HEARING LOSS

[For participants who reported experience with students with hearing difficulty on REDCap survey]

You mentioned on the survey that you've had experience with a student or students with hearing difficulty.

- Can you describe your experience?

HEARING SCREENING AND FOLLOW-UP PROCESS

[For participants who reported experience with school hearing screening and/or follow-up on REDCap survey]

You reported that you have experience with school hearing screening and/or follow-up.

- Can you walk me through from start to finish the process of screening students' hearing in your district? *(Looking for input on who, when, where, how, why; Who screens, equipment used, how screening is done, how results are communicated and to who, as well as any policy or school requirements followed; also need specific details on parental permission of health screenings in schools)*
- What are some challenges you've experienced with current or past hearing screening and/or follow-up processes?

TRIAL-SPECIFIC QUESTIONS

Appalachian STAR Trial Model

<u>Narrative Intro:</u> [Show or explain image of enlarged graphic.] There are 4 parts to the model we would like to adapt and implement for Kentucky. We are calling this the Appalachian STAR Trial. "STAR" stands for Specialty Telemedicine Access for Referrals. [Explains each of the components]. We would like your feedback on each of these 4 areas.

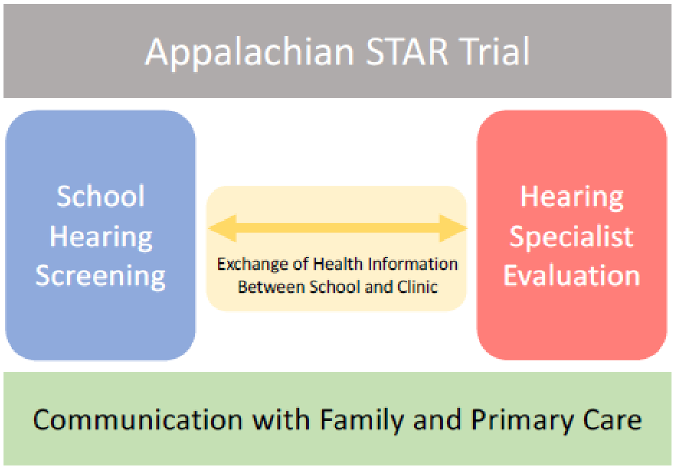

- Before we get into the details related to this model, are there other similar follow-up programs in your school/clinic/office? If so, how do they compare?

CORE COMPONENT: School Hearing Screening

- What are the most important challenges that we should anticipate and address within the school hearing screening process?
- Who are the key individuals to involve with the new screening process?

CORE COMPONENT: Hearing Specialist Evaluation

<u>Narrative Intro</u>: We want to look at how well the telehealth follow-up would work if performed in the school, maybe by school staff rather than a medical clinic off-site. This follow-up would be done shortly after the first hearing screening. It would include getting some additional information, such as images of

the ears. This information would be sent to an audiologist or hearing healthcare specialist for review and care plan.

[For participants who reported experience with telehealth on REDCap survey]

You mentioned on the survey that you've had experience with telehealth.

- Can you describe your experience?

[For all participants]

- How could we ensure that this model is feasible within your current workload? (Probe: How might this school-based telehealth model integrate into your everyday schedule?)
- What are some potential challenges or barriers to sending telehealth consults from the school to the consulting audiologist?

CORE COMPONENT: Exchange of Health Information Between School & Specialty Clinic/Provider

Narrative Intro: *Our work in rural Alaska showed students and families don't always get the information they need for follow up hearing care. At the same time, we noticed that hearing specialists had a hard time communicating directly with the school or teachers regarding a student's needs. We want to improve communication, so kids don't fall through the cracks. The next few questions ask you about improving this communication.*

- Generally, what kind of information exchange does your school have with clinics/medical offices in your community?
- How do the appropriate clinical personnel learn about whether or not students need follow-up after a school hearing screening? (Probe: parent/guardian contacts clinic, interoffice memo between school and clinic, ROI between school/clinic, letter, phone call, etc.)
- When thinking about communication between the school and the clinics/medical offices, what challenges might we encounter? How could we avoid communication breakdowns? *(probe: when, how, what should be shared; what should this exchange look like)*

CORE COMPONENT: Communication with Family and Primary Care

- How does the school share health information with families? With primary care providers? What works best?
- When thinking about communication with families and primary care providers, what challenges might we encounter? How could we avoid communication breakdowns? *(probe: when, how, what should be shared; what should this exchange look like; previous work suggests text/email)*

OVERALL Appalachian STAR Trial Model

- In summary, when thinking about the four main parts of the Appalachian STAR model, what you said would work for your clinic/office right now was _______________? What you said might not work is _________________? Is that an accurate summary?
    - Is there anything else you would like to share that we haven't yet discussed?

- Who else should we talk with?

Appalachian STAR Trial

<u>Parent Stakeholder Interview Questions</u>

INTERVIEWER-BACKGROUND, EXPECTATIONS & INTRODUCTIONS

<u>Narrative Intro:</u> Childhood hearing loss has lifelong consequences. Hearing screening in schools is an important start to identification and treatment. A common problem is follow-up from school hearing screenings: many children who are screened at school and referred to a hearing specialist do not receive this follow-up care. In rural Kentucky, it can be particularly difficult for parents and families to get follow-up care because hearing specialists can be hard to find.

We are interested in bringing follow-up into the school where the children already are. This is possible with telehealth, a technology that allows for virtual visits with hearing specialists. We did something similar and found it successful in rural Alaska.

We want to know from parents like you about how this model of school hearing screening and telehealth follow-up with a specialist could work in Kentucky schools. Hearing your perspective can help ensure we are setting up this program for success locally.

Thanks for sharing a little bit about yourself in the initial survey! It looks like you have children attending __________ school. We're looking forward to talking to today about school hearing screenings and follow-up.

EXPERIENCE WITH CHILDHOOD HEARING LOSS

[For participants who reported experience with hearing screenings]

You mentioned on the survey that you've had experience with the school hearing screening.

- Can you describe your experience?

HEARING SCREENING AND FOLLOW-UP PROCESS

[For participants who reported receiving information from the school on REDCap survey]

You reported you have received information from the school regarding your child's hearing screening results?

- Can you describe the information you received? [Probe: how, what, when, who; what did information mean to parent]

[For participants who reported taking child to the doctor for ear/hearing issue on REDCap survey]

You reported taking your child to the doctor for an ear or hearing concern.

- Was this as a follow-up from a school hearing screening?
- Can you describe the follow-up experience [probe: provider type, facility, testing, results, outcome of referral]
- What have been the challenges to receiving ear and hearing care for your child?

TRIAL-SPECIFIC QUESTIONS

Appalachian STAR Trial Model

*<u>Narrative Intro:</u> [Show or explain image of enlarged graphic.] There are 4 parts to the model we would like to adapt and implement for Kentucky. We are calling this the Appalachian STAR Trial. "STAR" stands for Specialty Telemedicine Access for Referrals***.** *[Explains each of the components]. We would like your feedback on each of these 4 areas.*

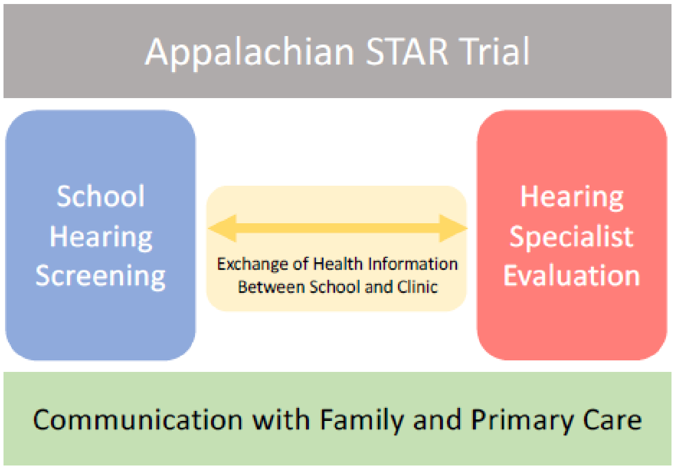

- Before we get started, are there other similar programs that you know of in your school/district? If so, how do they compare?

CORE COMPONENT: School Hearing Screening

<u>Narrative Intro:</u> Though school hearing screenings *are recommended, they are inconsistently implemented across the US, including here in Kentucky.*

- What do you think some challenges are within school hearing screenings?

CORE COMPONENT: Hearing Specialist Evaluation

<u>Narrative Intro</u>: We want to look at how well the telehealth would work if we used it on site at the school between school staff and a hearing specialist. This would involve getting some additional information from the child, such as taking images of the ears. This information would be sent to the audiologist or hearing healthcare specialist, who would then develop a care plan with the child and family via telehealth.

- As a parent, what are your thoughts on this process? *(Probe: Benefits? Concerns? Seek to understand parents' preferences on to be involved in this follow-up)*

<u>*Narrative Intro:*</u> *In school hearing screenings, poor communication between schools, clinical providers, and families can be part of the reason for the loss to follow up. We want to improve these communications, so kids don't fall through the cracks. The next few questions will ask you about improving this communication.*

CORE COMPONENT: Communication with Family and Primary Care

- What is your experience with how the school shares health information with you as the child's family? How about with primary care providers regarding your child's screening results? *(Probes?____________)*
- When thinking about communication with families, such as yourself, and primary care providers, what challenges might we encounter? How could we avoid communication breakdowns? *(probe: when, how, what should be shared; what should this exchange look like; previous work suggests text/email)*

CORE COMPONENT: Exchange of Health Information Between School & Specialty Clinic/Provider

- What is your experience with how school personnel (e.g. SLP, teacher, special education) and clinical/medical providers communicate regarding referred school hearing screening and any follow-up? (Prompt: interoffice memo, ROI between school/clinic, letter, phone call, etc.)
- When thinking about communication between the school and clinical/medical providers, what challenges might we encounter? How could we avoid communication breakdowns? *(probe: when, how, what should be shared; what should this exchange look like)*

- **OVERALL Appalachian STAR Trial Model**
- In summary, when thinking about the four main parts of the Appalachian STAR model, what you said were benefits were _____________ and potential challenges were ______________ . Is that an accurate summary?
    - Is there anything else you would like to share that we haven't yet discussed?

- Who else should we talk with?
