# Supplementary material for: Improving Access to Specialty Care for Rural Children Using Enhanced Hearing Screening and Specialty Telehealth Follow-Up in Rural Kentucky Schools: Protocol for a Hybrid Effectiveness-Implementation Stepped Wedge, Cluster-Randomized Controlled Trial (Appalachian STAR Trial)
Source: JMIR Res Protoc. 2025 Aug 26;14:e77630. doi: 10.2196/77630 (PMC12421207; doi:10.2196/77630)
Supplement: Multimedia Appendix 3 [file resprot_v14i1e77630_app3.pdf]

SUMMARY STATEMENT

(Privileged Communication)

PROGRAM CONTACT:
Dr Cheryl Boyce
301-435-1070
cboyce@nih.gov

Release Date: 08/16/2021
Revised Date:

Application Number: 1 U01 DE031539-01

Principal Investigators (Listed Alphabetically):

BUSH, MATTHEW LEE
EMMETT, SUSAN DAVIS (Contact)

Applicant Organization: DUKE UNIVERSITY

Review Group: ZRG1 MOSS-T (50)
Center for Scientific Review Special Emphasis Panel
RFA-RM-21-021: UNITE Transformative Research to Address Health Disparities and Advance Health Equity (U01)

Meeting Date: 07/27/2021
Council: AUG 2021
Requested Start: 09/01/2021
RFA/PA: RM21-021
Dual IC(s): RM, OD

Project Title: Appalachian STAR Trial

SRG Action: Impact Score:19
Next Steps: Visit https://grants.nih.gov/grants/next_steps.htm
Human Subjects: 30-Human subjects involved - Certified, no SRG concerns
Animal Subjects: 10-No live vertebrate animals involved for competing appl.
Gender: 1A-Both genders, scientifically acceptable
Minority: 1A-Minorities and non-minorities, scientifically acceptable
Age: 1A-Children, Adults, Older Adults, scientifically acceptable

Project Year	Direct Costs Requested	Estimated Total Cost
1	600,313	950,473
2	665,806	1,054,168
3	759,152	1,201,962
4	727,610	1,152,022
5	745,011	1,179,573
TOTAL	3,497,892	5,538,199

1U01DE031539-01 Emmett, Susan

RESUME AND SUMMARY OF DISCUSSION: The goal of this U01 application is to evaluate a novel model of school-based telehealth-driven preventive care (STAR) in Appalachian schools of rural eastern Kentucky to reduce loss to follow-up from school hearing screening and improve access to specialty care in rural environments. Reviewers noted that the study is highly significant and can be applied to improve access to other preventive health care services and referral to specialty care for children living in rural areas across the country. The multidisciplinary investigative team has strong expertise in implementation science, audiology, health policy, and epidemiology. Thus, they are well positioned to successfully complete the proposed studies. The team would adapt their prior work experience to the proposed studies in rural Kentucky, which further establishes feasibility. Engaging schools with public health care and the involvement of community stakeholders throughout the study are additional strengths. Additional strengths include a community centered approach, including school administrators, teachers, audiologists, parents, and a strong connection with State leadership with the potential to implement policy changes. The study design is strong which includes a stepped-wedge, cluster randomized hybrid type-1 effectiveness-implementation trial across 66 schools in 14 rural Kentucky counties. The mixed method evaluation of the implementation process using Consolidated Framework of Implementation Research domains is well developed. However, some minor weaknesses were identified. Reviewers noted that there is no discussion on cost-effectiveness measures and school funding for schools and health Cafes as potential barriers to broad implementation are not addressed. The application aims to bring telehealth technology directly to schools but there is insufficient detail to understand if the entire process can be done reliably and accurately by school staff. Fidelity assessment has not been sufficiently addressed and the timeline may not allow for full testing of this model. Stakeholder engagement of superintendents is only at the advisory level. Nonetheless, reviewers noted that these weaknesses are addressable and minor. Thus, the overall enthusiasm of the review panel remained extremely high for this exceptional application.

DESCRIPTION (provided by applicant): Health disparities in rural America begin early in life, arising from social determinants of health that start in childhood. School health programs often provide the only access to preventive services for rural children. However, school screening is variably implemented, plagued by loss to follow-up, and limited specialists in rural areas compound barriers to care. We propose to prospectively implement a novel model of care in Appalachian schools of rural Kentucky to address social determinants at the school, health system, and policy levels that hinder identification and treatment of preventable health disparities for two NIH-designated disparity populations: underserved rural and socioeconomically disadvantaged children. Our goal is to establish a novel, generalizable model of school-based, telehealth-driven preventive care that can be disseminated in underserved populations across rural America. We will adapt and evaluate our evidence-based approach, “STAR” (Specialty Telemedicine Access for Referrals), that we have found effective in a Tribal setting in rural Alaska. Appalachia has some of the poorest counties in the US, making this region ideal for adapting across rural America. The innovative “Appalachian STAR trial” will be the first study to apply school-based telehealth for preventive services, with direct access to specialists. Hearing screening will be the prototype for STAR due to the high burden of preventable, infection-related hearing loss in underserved children and the profound lifelong implications of childhood hearing loss. Our interdisciplinary team has relationships with underserved communities in Kentucky and partnership with a Community Advisory Board and Stakeholder Advisory Board providing support from the highest levels of state government (See KY Governor Letter). We will begin by adapting the STAR model of care to meet the needs of rural communities and schools through a community- and stakeholder-driven approach. We will evaluate the STAR care model in 66 schools in rural Kentucky through a stepped wedge cluster-randomized hybrid type 1 effectiveness-implementation trial with kindergarten children in 14 counties (n=~3600/year). The STAR intervention includes county-level school screening policy change with enhanced mHealth school hearing screening, followed by virtual specialty care referral. The stepped-wedge design allows evaluation of the policy/screening and referral components as well as comparison

of usual care vs. full intervention (years 2 vs.5), while meeting community input that the intervention be available to all. Primary outcomes are the percentage of 1) children screened and 2) referrals resulting in specialty care within two months of screening. We conservatively hypothesize the percentage screened will improve by 20% and follow-up will improve by 40%. During the trial, we will assess multi-level implementation factors and outcomes to inform scale- up into other rural areas. Our STAR model could be both scaled across rural America and applied to other preventable health disparities, combining policy change on school health with digital innovations to radically expand access to care for underserved rural and socioeconomically disadvantaged children nationwide.

PUBLIC HEALTH RELEVANCE: School-based health programs often provide the only access to preventive services for underserved rural children, but loss to follow-up and limited numbers of specialists are problematic. This study will prospectively evaluate a new model of school-based, telehealth-driven preventive care (STAR) in Appalachian schools of rural eastern Kentucky to reduce loss to follow-up from school hearing screening and improve access to specialty care in rural environments. This telehealth-driven model could be applied to a wide range of preventable health disparities and translated to other underserved populations across rural America.

CRITIQUE 1

Significance: 1
Investigator(s): 1
Innovation: 2
Approach: 2
Environment: 1

Overall Impact: This proposal addresses the urgent need to improve access to preventive health care services and referral to specialty care for children living in rural areas. The team will establish and test a novel, generalizable model of school-based, telehealth-driven preventive care that can be disseminated and implemented in underserved settings across rural America. The team focuses on prevention of hearing loss among children in rural areas (60% of hearing loss is preventable), based on their successful model working with tribal communities in Alaska, as a test case for this new model of care.

This is a very strong team well positioned to adapt their prior work to the proposed setting in rural Kentucky. They propose a strong study design including a stepped wedge, cluster randomized hybrid type 1 effectiveness-implementation trial design. They will use approach (ADAPT-ITT) to adapt the original model for STAR (Specialty Telemedicine Access for Referrals) test by this team in Alaska for current study. There is a well-developed plan for mixed method evaluation of the implementation process using Consolidated Framework of Implementation Research domains, making use of coinvestigators with expertise in both quantitative and qualitative methods. Community and stakeholder input is incorporated throughout.

Weaknesses are relatively minor. This is no discussion of health care and school funding as potential barriers to broad implementation; cost and cost-effectiveness measures are not planned. In addition, the model relies on the availability of referral specialty clinics, potentially limiting implementation in some settings.

Overall, if successful this new model of care has the potential to transform access to needed health care for a vulnerable population of children living in rural areas. Impact on preventable child health problems would improve health and educational outcomes for their lifespan. This new system of care could be adapted to meeting the needs of vulnerable populations in other geographic settings and thereby transform health care for many communities living with health disparities.

1. Significance:

Strengths

- Addresses the urgent issue of access to preventive health care services and referral to specialty care for children living in rural areas.
- Works by engaging schools with public health and health care, creating systems change toward efficiency and with substantial population reach.
- If successful in this case and setting the approach has the potential to transform health care for children living in rural counties of the US.
- Focus of this study (test case) is on prevention of children's hearing loss, a crucial medical issue that has long-term consequences for child health and educational achievement, that is highly impacted by access to healthcare.

Weaknesses

- None noted.

2. Investigator(s):

Strengths

- Very strong interdisciplinary team with expertise including implementation science, audiology, health policy and epidemiology.
- The Duke PI is an otolaryngologist with public health and global heath expertise, policy expertise, and experience conducting research using telehealth in Alaska.
- The Kentucky PI is also an otolaryngologist who research is focused on increasing access to and improving timely delivery of healthcare in underserved populations
- The team has experience with these types of intervention through their work in rural Alaska.

Weaknesses

- None noted.

3. Innovation:

Strengths

- Integrates telemedicine into schools across rural communities that can be adapted and used across the United States.
- Tests a new model, built on the team's experience in Alaska, that will be applicable to other health care needs in rural areas and can be rapidly disseminated and scaled up by their existing national research network.
- Addresses systems barriers by better linking kids to the specialty care clinics that the investigators show are underutilized.
- A strong Community Advisory Board provides high level support, including from the Governor of KY
- Outcome measures go beyond initial screening and recognizes the importance of completed referrals to specialty care.

Weaknesses

- The model relies on the availability of referral specialty clinics, potentially limiting implementation in some settings.

4. Approach:

Strengths

- Strong study design including a stepped-wedge cluster randomized hybrid type 1 effectiveness/implementation trial across 66 schools in 14 rural Kentucky counties.
- Outcomes include both screening and referral.
- Using an established approach (ADAPT-ITT) to adapt the original model for STAR (Specialty Telemedicine Access for Referrals) in Alaska, for current study including engagement of education, parent and health care stakeholders.
- Well-developed plan for mixed method multi-level evaluation of the implementation process using Consolidated Framework of Implementation Research domains, making use of coinvestigators with expertise in both quantitative and qualitative methods.
- Community and stakeholder input is incorporated throughout.
- Tests the intervention in 14 counties to create generalizable model of school-based telehealth-driven preventive care

Weaknesses

- No discussion of health care and school funding as potential barriers to broad implementation
- Cost and cost-effectiveness measures would add to the value and potential acceptability.

5. Environment:

Strengths

- Outstanding environment for this study in a high need rural state with a local PI and strong support from state leadership.
- Letters of support from the superintendents in the 14 participating counties as well as the governor show strong support for testing this new care model. This will enable needed policy and systems changes.

Weaknesses

- None noted.

Study Timeline:

Strengths

- Timeline for activities is well specified.
- Timeline is feasible given the experience of the team and the already established commitments.

Weaknesses

- None noted.

Protections for Human Subjects

Acceptable Risks and/or Adequate Protections

Data and Safety Monitoring Plan (Applicable for Clinical Trials Only):

Acceptable

- There is no proposed DSMB.

Inclusion Plans

- Sex/Gender: Distribution justified scientifically
- Race/Ethnicity: Distribution justified scientifically
- For NIH-Defined Phase III trials, Plans for valid design and analysis: Not applicable
- Inclusion/Exclusion Based on Age: Distribution justified scientifically
- All are appropriate

Vertebrate Animals

Not Applicable (No Vertebrate Animals)

Biohazards

Not Applicable (No Biohazards)

Resource Sharing Plans

Acceptable

Authentication of Key Biological and/or Chemical Resources

Not Applicable (No Relevant Resources)

Budget and Period of Support

Recommend as Requested

CRITIQUE 2

Significance: 1
Investigator(s): 1
Innovation: 1
Approach: 3
Environment: 2

Overall Impact: The goal of the proposal is to establish a novel, generalizable model of school-based, telehealth-driven hearing screenings that can be disseminated to other preventive services as well as to underserved populations across rural America. The proposal addresses 2 topics of high importance: rural health disparities in preventive care and childhood hearing loss. It also utilizes telehealth strategies which are timely and relevant following the rapid expansion of telehealth services during the COVID-19 pandemic. The proposal highlights how the current project will extend relevant prior work

completed by the study team and overcome challenges identified in those studies. Strengths include experienced research team with excellent community/stakeholder engagement in multiple phases of the project, a highly innovative intervention which focuses on school-based telehealth preventive services, and appropriate study design informed by relevant conceptual frameworks. Weaknesses in the approach are mainly concerning feasibility given the lack of preliminary data on the efficacy, accuracy, or reliability of the proposed telehealth intervention being delivered by school "lay" staff and the need for clarification on how different the intervention is from current standard care (to better understand training and adaptation needs). The proposal has high transformative potential: if effective, the STAR model could be both scaled across rural America and applied to other preventable health disparities.

1. Significance:

Strengths

- The proposal addresses 2 topics of high importance: rural health disparities in preventive care and childhood hearing loss.
- The proposal utilizes telehealth strategies which are timely and relevant following the rapid expansion during the COVID-19 pandemic.
- The proposal has transformative potential: if effective, the STAR model could be both scaled across rural America and applied to other preventable health disparities.
- The proposal includes preliminary data from the investigators' prior projects among tribal communities in Alaska and highlights how the current project will extend that work and overcome challenges identified in those studies.

Weaknesses

- None noted by reviewer.

2. Investigator(s):

Strengths

- Excellent team of investigators with expertise and experience in relevant research and clinical topics.
- Collaboration plan and organization of study team is appropriate for the proposed work.

Weaknesses

- None noted by reviewer.

3. Innovation:

Strengths

- Highly innovative: "Appalachian STAR trial" will be the first study in the nation to apply school-based telehealth for preventive services, directly connecting rural teachers and school nurses to specialists.

Weaknesses

- None noted by reviewer.

4. Approach:

Strengths

- Proposal includes a strong framework for the adaptation phase, ADAPT-ITT, with good rationale for its use.
- Effective study design selection for aim 2 with a stepped wedge, cluster randomized Hybrid type 1 effectiveness-implementation trial of the STAR model of care.
- Appropriate effectiveness outcome measures for Aim 2 that are readily accessible in available data; power and sample size calculations are informed by preliminary work of the study team.
- Selection of implementation measures for Aim 3 guided by strong framework (CFIR).
- Appropriate inclusion of relevant biological variables.

Weaknesses

- Review of prior research/prelim data justifies the plan to bring telehealth technology directly to schools but not enough detail to understand if the entire process can be done reliably and accurately by school staff. The proposal states “telehealth equipment designed for lay-person use” will be involved but prior research review does not explain how it has been designed for lay-person use, if it has undergone validity and reliability testing in lay settings, etc. The potential challenges acknowledge this issue with the testing described in Aim 1 being the solution; however, doing this work may go beyond the scope of a single Aim and significantly impact ability of the team to proceed with subsequent Aims.
- Feasibility concerns: Difficult to understand how much work needs to be done in terms of intervention adaptation and training of staff without a detailed comparison (table or figure) of standard screening versus mHealth pure-tone screening with tympanometry + telehealth referral as well as more information about the new technology (devices, procedures to use them, how they differ from current standards).
- If accuracy and reliability of testing is operator dependent (particularly with initial roll out of the intervention), the inability to review results in “real-time” (due to asynchronous telehealth model) could create significant challenges (need for repeat testing, informing parents of results, etc.).

5. Environment:

Strengths

- Excellent institutional support and state-level support to conduct the proposed study.
- Excellent community engagement with LOS from all county superintendents.

Weaknesses

- The telehealth equipment necessary for the intervention is only described in the budget/budget justification. Additional details about it would be helpful in the equipment section or the research strategy to better understand the training, resources, etc. necessary to utilize the new technology.

Study Timeline:

Strengths

- Timeline is sufficiently detailed.

Weaknesses

- Potential challenges in completing Aim 1 could affect the rest of the timeline but contingency plans are not included in the timeline.

Protections for Human Subjects

Acceptable Risks and/or Adequate Protections

Data and Safety Monitoring Plan (Applicable for Clinical Trials Only):

Acceptable

Inclusion Plans

- Sex/Gender: Distribution justified scientifically
- Race/Ethnicity: Distribution justified scientifically
- For NIH-Defined Phase III trials, Plans for valid design and analysis: Not applicable
- Inclusion/Exclusion Based on Age: Distribution justified scientifically

Vertebrate Animals

Not Applicable (No Vertebrate Animals)

Biohazards

Not Applicable (No Biohazards)

Resource Sharing Plans

Acceptable

Authentication of Key Biological and/or Chemical Resources

Not Applicable (No Relevant Resources)

Budget and Period of Support

Recommend as Requested

CRITIQUE 3

Significance: 1
Investigator(s): 1
Innovation: 2
Approach: 2
Environment: 1

Overall Impact: School-based health programs often provide the only access to preventive services for underserved rural children, but loss to follow-up and limited numbers of specialists are problematic. This study will prospectively evaluate a new model of school-based, telehealth-driven preventive care

(STAR) in Appalachian schools of rural eastern Kentucky to reduce loss to follow-up from school hearing screening and improve access to specialty care in rural environments. This telehealth-driven model could be applied to a wide range of preventable health disparities and translated to other underserved populations across rural America.

This study will adapt and evaluate “STAR” (Specialty Telemedicine Access for Referrals), that has been found effective in a Tribal setting in rural Alaska. Appalachia has some of the poorest counties in the US, making this region ideal for adapting across rural America. The innovative “Appalachian STAR trial” will be the first study to apply school-based telehealth for preventive services, with direct access to specialists. Hearing screening will be the prototype for STAR due to the high burden of preventable, infection-related hearing loss in underserved children and the profound lifelong implications of childhood hearing loss. Primary outcomes are the percentage of 1) children screened and 2) referrals resulting in specialty care within two months of screening.

The study links school screening to specialty care for a condition that is both serious, consequential and treatable.

Major Strengths: This is a scientifically outstanding proposal by an exceptional team of experts experienced in otology and implementation and dissemination methods who are addressing a critical issue in an underserved population.

Major Weaknesses: There are no major weaknesses in this proposal.

Transformative Impact: While there are many discussions about how to increase the scale of access to health services, we have lacked in a model of how this can be done. This protocol offers a substantial model to offer increased access to a group of children who would otherwise go untreated.

1. Significance:

Strengths

- Addresses a critical, treatable issue of screening for hearing in Appalachia.
- Has a design and impact that can truly transform screening and increase access.
- Well-designed study and meaningful endpoints that benefit both the study participants and students in our nation who have been previously left behind.
- Step-wedge design provides screening and treatment and a robust clinical trial.
- Transformative Impact—while there are many discussions about how to increase the scale of access to health services, we have lacked in a model of how this can be done. This protocol offers a substantial model to offer increased access to a group of children who would otherwise go untreated.

Weaknesses

- None noted.

2. Investigator(s):

Strengths

- Susan Emmett, MD, MPH, Contact PI and Associate Professor at Duke University, is an NIH-funded otolaryngology surgeon scientist with policy expertise who conducts hearing loss disparities research in underserved populations. Dr. Emmett is Director of HEAR-USA and Co-Chair of Innovations in Service Delivery for the Lancet Commission on Hearing Loss. She co-led the PCORI trial and will provide overall project leadership.

- Matthew Bush, MD, PhD, Multiple PI, SAB Director, and Professor and Vice Chair at University of Kentucky, is an otolaryngology surgeon scientist with expertise in implementation science and rural health disparities. He Co-Directs HEAR-USA and collaborates closely with Kentucky state leadership as PI of an NIDCD R01 on access to care for newborns with hearing loss.
- Dr. Bush will lead the SAB and oversee STAR implementation in schools. Janet Bettger, PhD, Co-Investigator and Associate Professor at Duke, is a health services researcher and implementation scientist with expertise in cluster randomized trials and developing evidence on care models to inform policy change.
- Dr. Bettger will oversee the implementation evaluation of the STAR model.
- Nancy Schoenberg, PhD, Co-Investigator, is the founding Director of the Center for Health Equity Transformation and Associate Vice President for Research on Health Disparities at University of Kentucky. She has led multiple NIH-funded studies and brings expertise in community engagement and mixed methodology to the study team.
- Lori Travis, AuD, Co-Investigator and SAB Member, is the Speech and Hearing Administrator for Kentucky's Office for Children with Special Health Care Needs (OCSHCN) and a Co-I on Dr. Bush's R01. She will lead implementation of STAR in OCSHCN clinics.
- Elizabeth Turner, PhD, Co-Investigator and Lead Statistician, is Professor and Director of the Duke Global Health Institute Research Design and Analysis Core. She has been the lead statistician on 14 cluster-randomized trials, including the Alaska PCORI trial, and is Co-Investigator on a PCORI methods grant on stepped wedge designs.
- Samantha Robler, PhD, AuD, Consultant and Key Personnel, is a telemedicine expert, audiologist, and researcher for Norton Sound Health Corporation in rural Alaska. She co-led the PCORI trial with Dr. Emmett and will oversee STAR telehealth integration into schools.
- Tina Studts, PhD, Consultant and Key Personnel, is Associate Professor at University of Colorado. She is an implementation scientist who brings nationally recognized expertise in ADAPT-ITT and will oversee adaptation of the STAR model for rural Kentucky.
- The investigators have the necessary experience to conduct a clinical trial, manage data and support engagement of the School District, which is their community.

Weaknesses

- The commissioner of Education and the Secretary of Health might strengthen the key personnel as part of the study team instead of just consultants.

3. Innovation:

Strengths

- Shifts the paradigm for screening plus referral plus services.
- Could provide access and care for thousands of children within the study itself and change the course of lives while advancing D and I research.

Weaknesses

- None noted.

4. Approach:

Strengths

- Study design is a well-described step-wedge approach.
- Approach ensures a robust and unbiased approach.
- Biological variables are accounted for, trial is inclusive.
- Rigor and reproducibility is appropriate and thoughtfully constructed.
- Data collection and analysis is well-described, appropriate and has the requisite expertise.
- Design and sampling are well-justified.
- Study has been modified to fit into the allotted time-period.
- Community Engagement is robust through the school-district.
- Intervention is appropriate, step-wedge design so that all who participate will receive care.

Weaknesses

- Even more community engagement could result from working with PTA's, parents, teachers and children, thereby increasing trust and transparency in the work and in providers and districts.

5. Environment:

Strengths

- Participating academic institutions (University of Kentucky and Duke University) have outstanding resources for large-scale disparities studies (see Letters of Support). Both M-PIs have conducted cluster randomized trials in underserved populations.
- The Data Coordinating Center at the Duke Global Health Institute has extensive experience with large, randomized trials in vulnerable and hard-to-reach populations.
- An existing program in Kentucky for free audiology care at OCSHCN clinics will facilitate implementation of the STAR model. Through this program, children who receive referrals from school hearing screening programs can currently receive a diagnostic audiology evaluation at an OCSHCN clinic free of charge, but the program is poorly utilized.
- The STAR model has the potential to transform utilization of this service by removing barriers that result in loss to follow-up after school screening. This concept of regionally located, complementary specialty care connected to rural schools through our STAR model could be replicated elsewhere.
- Strong collaborations with state government provide unique opportunities for policy change and sustainable adoption of the STAR model.
- Strong support from School superintendents.

Weaknesses

- There are no environmental weaknesses.

Study Timeline:

Strengths

- Timeline is detailed and appropriate.

Weaknesses

- None noted.

Protections for Human Subjects
Acceptable Risks and/or Adequate Protections
Data and Safety Monitoring Plan (Applicable for Clinical Trials Only):
Acceptable

Inclusion Plans

- Sex/Gender: Distribution justified scientifically
- Race/Ethnicity: Distribution justified scientifically
- For NIH-Defined Phase III trials, Plans for valid design and analysis: N/A
- Inclusion/Exclusion Based on Age: Distribution justified scientifically

Vertebrate Animals
Not Applicable (No Vertebrate Animals)

Biohazards
Not Applicable (No Biohazards)

Resource Sharing Plans
Acceptable

Authentication of Key Biological and/or Chemical Resources
Not Applicable (No Relevant Resources)

Budget and Period of Support
Recommend as Requested

THE FOLLOWING SECTIONS WERE PREPARED BY THE SCIENTIFIC REVIEW OFFICER TO SUMMARIZE THE OUTCOME OF DISCUSSIONS OF THE REVIEW COMMITTEE, OR REVIEWERS' WRITTEN CRITIQUES, ON THE FOLLOWING ISSUES:

PROTECTION OF HUMAN SUBJECTS: ACCEPTABLE

INCLUSION OF WOMEN PLAN: ACCEPTABLE

INCLUSION OF MINORITIES PLAN: ACCEPTABLE

INCLUSION ACROSS THE LIFESPAN: ACCEPTABLE

COMMITTEE BUDGET RECOMMENDATIONS: The budget was recommended as requested.

Footnotes for 1 U01 DE031539-01; PI Name: Emmett, Susan Davis

NIH has modified its policy regarding the receipt of resubmissions (amended applications).See Guide Notice NOT-OD-18-197 at https://grants.nih.gov/grants/guide/notice-files/NOT-OD-18-197.html. The impact/priority score is calculated after discussion of an application by averaging the overall scores (1-9) given by all voting reviewers on the committee and multiplying by 10. The criterion scores are submitted prior to the meeting by the individual reviewers assigned to an application, and are not discussed specifically at the review meeting or calculated into the overall impact score. Some applications also receive a percentile ranking. For details on the review process, see http://grants.nih.gov/grants/peer_review_process.htm#scoring.

MEETING ROSTER

Center for Scientific Review Special Emphasis Panel
CENTER FOR SCIENTIFIC REVIEW
RFA-RM-21-021: UNITE Transformative Research to Address Health Disparities and Advance Health Equity (U01)
ZRG1 MOSS-T (50)
07/27/2021 - 07/28/2021

Notice of NIH Policy to All Applicants: Meeting rosters are provided for information purposes only. Applicant investigators and institutional officials must not communicate directly with study section members about an application before or after the review. Failure to observe this policy will create a serious breach of integrity in the peer review process, and may lead to actions outlined in NOT-OD-14-073 at https://grants.nih.gov/grants/guide/notice-files/NOT-OD-14-073.html, NOT-OD-15-106 at https://grants.nih.gov/grants/guide/notice-files/NOT-OD-15-106.html, and NOT-OD-18-115 at https://grants.nih.gov/grants/guide/notice-files/NOT-OD-18-115.html, including removal of the application from immediate review.

CHAIRPERSON(S)

MURRY, VELMA MCBRIDE, PHD
LOIS AUTREY BETTS CHAIR AND JOE B WYATT
DISTINGUISHED UNIVERSITY PROFESSOR
DEPARTMENT OF HUMAN AND ORGANIZATIONAL DEVELOPMENT
VANDERBILT UNIVERSITY
NASHVILLE, TN 37203

MEMBERS

ABRAIDO-LANZA, ANA F., PHD
PROFESSOR
VICE DEAN SOCIAL AND BEHAVIORAL SCIENCES
SCHOOL OF GLOBAL PUBLIC HEALTH
NEW YORK UNIVERSITY
NEW YORK, NY 10012

ADUNYAH, SAMUEL E, PHD
PROFESSOR AND CHAIRMAN
DEPARTMENT OF BIOCHEMISTRY, CANCER BIOLOGY
NEUROSCIENCE AND PHARMACOLOGY
SCHOOL OF MEDICINE
MEHARRY MEDICAL COLLEGE
NASHVILLE, TN 37208

ARORA, KAVITA SHAH, MD
ASSOCIATE PROFESSOR
METROHEALTH MEDICAL CENTER
CASE WESTERN RESERVE UNIVERSITY
CLEVELAND, OH 44109

BENTLEY-EDWARDS, KEISHA L., PHD
ASSOCIATE DIRECTOR OF RESEARCH, SAMUEL DUBOIS
COOK CENTER ON SOCIAL EQUITY
DUKE UNIVERSITY
DURHAM, NC 27708

BORREGO, MATTHEW, PHD
PROFESSOR
DEPARTMENT OF PHARMACY PRACTICE
AND ADMINISTRATIVE SCIENCES
COLLEGE OF PHARMACY
UNIVERSITY OF NEW MEXICO
ALBUQUERQUE, NM 87131

BRUCE, MARINO A, PHD
CLINICAL PROFESSOR OF BEHAVIORAL AND SOCIAL SCIENCES
COLLEGE OF MEDICINE
UNIVERSITY OF HOUSTON
HOUSTON, TX 77004

BURKE, NANCY J., PHD
DEPARTMENT CHAIR AND PROFESSOR
DEPARTMENT OF PUBLIC HEALTH
SCHOOL OF SOCIAL SCIENCES AND HUMANITIES
UNIVERSITY OF CALIFORNIA, MERCED
MERCED, CA 95343

CASSIDY-BUSHROW, ANDREA E, PHD
ASSOCIATE SCIENTIST AND RESEARCH EPIDEMIOLOGIST
DEPARTMENT OF PUBLIC HEALTH SCIENCES
HENRY FORD HEALTH SYSTEM
DETROIT, MI 48202

CASTRO, EIDA MARIA, PSYD
ASSOCIATE PROFESSOR
DEPARTMENT OF PSYCHIATRY
MENTAL HEALTH DIVISION
SCHOOL OF BEHAVIORAL AND BRAIN SCIENCES
PONCE SCHOOL OF MEDICINE
PONCE, PR 00716

CHAKKALAKAL, ROSETTE J, MD
ASSOCIATE PROFESSOR
DIVISION OF GENERAL INTERNAL MEDICINE AND PUBLIC HEALTH
MEDICAL CENTER
VANDERBILT UNIVERSITY
NASHVILLE, TN 37235

CHATTERJI, PINKA, PHD
PROFESSOR
ECONOMICS DEPARTMENT
UNIVERSITY AT ALBANY
ALBANY, NY 12222

CHAVEZ, LIGIA M., PHD
ASSOCIATE PROFESSOR
BEHAVIORAL SCIENCES RESEARCH INSTITUTE
UNIVERSITY OF PUERTO RICO
RIO PIEDRAS, PR 00935

COHN, ELIZABETH GROSS, PHD
RUDIN CHAIR AND PROFESSOR OF COMMUNITY-ENGAGED RESEARCH
ASSOCIATE PROVOST FOR RESEARCH
HUNTER COLLEGE
CITY UNIVERSITY OF NEW YORK
NEW YORK, NY 10065

CUBBIN, CATHERINE, PHD
PROFESSOR AND ASSOCIATE DEAN FOR RESEARCH
STEVE HICKS SCHOOL OF SOCIAL WORK
UNIVERSITY OF TEXAS AT AUSTIN
AUSTIN, TX 78712

DALE, SANNISHA K., PHD
ASSOCIATE PROFESSOR
HEALTH DIVISION
DEPARTMENT OF PSYCHOLOGY
UNIVERSITY OF MIAMI CORAL GABLES
CORAL GABLES, FL 33146

DIAZ, VANESSA ASTRUD, MD
ASSISTANT PROFESSOR
DEPARTMENT OF FAMILY MEDICINE
MEDICAL UNIVERSITY OF SOUTH CAROLINA
CHARLESTON, SC 29425

EHRENTHAL, DEBORAH BETH, MD, MPH
PROFESSOR
DEPARTMENT OF OBSTETRICS & GYNECOLOGY
AND POPULATION HEALTH SCIENCES
UNIVERSITY OF WISCONSIN
SCHOOL OF MEDICINE AND PUBLIC HEALTH
MADISON, WI 53726

ERINOSHO, TEMITOPE O, PHD
ASSOCIATE PROFESSOR
DEPARTMENT OF APPLIED HEALTH SCIENCES
SCHOOL OF PUBLIC HEALTH
INDIANA UNIVERSITY BLOOMINGTON
BLOOMINGTON, IN 27599

GAMAREL, KRISTINE E, PHD
JOHN G. SEARLE ASSISTANT PROFESSOR
DEPARTMENT OF HEALTH BEHAVIOR
AND HEALTH EDUCATION
SCHOOL OF PUBLIC HEALTH
UNIVERSITY OF MICHIGAN
ANN ARBOR, MI 48109

GONZALEZ, CRISTINA M, MD
PROFESSOR
DEPARTMENT OF MEDICINE
MONTEFIORE MEDICAL CENTER
ALBERT EINSTEIN COLLEGE OF MEDICINE
BRONX, NY 10461

HALL, WILLIAM JAMES, PHD
ASSISTANT PROFESSOR
SCHOOL OF SOCIAL WORK
UNIVERSITY OF NORTH CAROLINA CHAPEL HILL
CHAPEL HILL, NC 27599

HICKEN, MARGARET TAKAKO, PHD
RESEARCH ASSOCIATE PROFESSOR
SURVEY RESEARCH CENTER
INSTITUTE FOR SOCIAL RESEARCH
UNIVERSITY OF MICHIGAN
ANN HARBOR, MI 48104

HIRSHFIELD, SABINA, PHD
PRINCIPAL RESEARCH SCIENTIST
DEPARTMENT OF MEDICINE
STAR PROGRAM
SUNY DOWNSTATE HEALTH SCIENCES UNIVERSITY
BROOKLYN, NY 11203

KATZ, MIRA L, PHD
PROFESSOR
DEPARTMENT OF HEALTH BEHAVIOR
AND HEALTH PROMOTION
COLLEGE OF PUBLIC HEALTH
OHIO STATE UNIVERSITY
COLUMBUS, OH 43210

KIM, DANIEL, MD, DRPH
ASSOCIATE PROFESSOR
DEPARTMENT OF HEALTH SCIENCES
BOUVE COLLEGE OF HEALTH SCIENCES
NORTHEASTERN UNIVERSITY
BOSTON, MA 02115

KUNIN-BATSON, ALICIA S, PHD
ASSISTANT PROFESSOR
DEPARTMENT OF PEDIATRICS
UNIVERSITY OF MINNESOTA MEDICAL SCHOOL
MINNEAPOLIS, MN 55414

LEONE, LUCIA A, PHD
ASSOCIATE PROFESSOR
DEPARTMENT OF COMMUNITY HEALTH AND HEALTH BEHAVIOR
SCHOOL OF PUBLIC HEALTH AND HEALTH PROFESSIONS
STATE UNIVERSITY OF NEW YORK AT BUFFALO
BUFFALO, NY 14214

LI, YUE, PHD
PROFESSOR OF PUBLIC HEALTH SCIENCES
DIRECTOR, HEALTH SERVICES RESEARCH & POLICY (HSRP)
DIVISION OF HEALTH POLICY AND OUTCOMES RESEARCH
DEPARTMENT OF PUBLIC HEALTH SCIENCES
UNIVERSITY OF ROCHESTER MEDICAL CENTER
ROCHESTER, NY 14642

MARTINEZ, MARIA ELENA, PHD
SAM M. WALTON ENDOWED CHAIR FOR CANCER RESEARCH
PROFESSOR AND ASSOCIATE DIRECTOR
POPULATION SCIENCES, DISPARITIES
AND COMMUNITY ENGAGEMENT
UC SAN DIEGO MOORES CANCER CENTER
LA JOLLA, CA 92093

MARTINEZ, PRISCILLA, PHD
ASSOCIATE SCIENTIST
ALCOHOL RESEARCH GROUP
PUBLIC HEALTH INSTITUTE
EMERYVILLE, CA 94608

MCDONOUGH, IAN, PHD
ASSOCIATE PROFESSOR
DEPARTMENT OF PSYCHOLOGY
COLLEGE OF ARTS AND SCIENCES
UNIVERSITY OF ALABAMA, TUSCALOOSA
TUSCALOOSA, AL 35487

MITCHELL, SUZANNE E, MD
ASSOCIATE PROFESSOR
DEPARTMENT OF FAMILY MEDICINE
SCHOOL OF MEDICINE
BOSTON UNIVERSITY
BOSTON, MA 02118

MITSIADES, NICHOLAS, MD, PHD
ASSOCIATE PROFESSOR
MEDICINE-HEMATOLOGY AND ONCOLOGY
DEPARTMENT OF MOLECULAR AND CELLULAR BIOLOGY
COLLEGE OF MEDICINE
BAYLOR COLLEGE OF MEDICINE
HOUSTON, TX 77030

MOSKOWITZ, JUDITH T, PHD
PROFESSOR
DEPARTMENT OF MEDICAL SOCIAL SCIENCES
FEINBERG SCHOOL OF MEDICINE
NORTHWESTERN UNIVERSITY
CHICAGO, IL 60611

ODERO-MARAH, VALERIE, PHD
PROFESSOR & ASSISTANT DIRECTOR OF RESEARCH
DEPARTMENT OF BIOLOGICAL SCIENCES
CENTER FOR CANCER RESEARCH AND THERAPEUTIC DEVELOPMENT
CLARK ATLANTA UNIVERSITY
ATLANTA, GA 30314

PRESS, VALERIE G, MD
ASSOCIATE PROFESSOR
DEPARTMENTS OF MEDICINE AND PEDIATRICS
UNIVERSITY OF CHICAGO
CHICAGO, IL 60637

RANGACHARI, PAVANI, PHD
PROFESSOR
DEPARTMENT OF INTERDISCIPLINARY HEALTH SCIENCES
DEPARTMENT OF FAMILY MEDICINE (MCG)
THE GRADUATE SCHOOL
AUGUSTA UNIVERSITY
AUGUSTA, GA 30912

RICKS-SANTI, LUISEL J., PHD
DIRECTOR
CANCER RESEARCH CENTER
HAMPTON UNIVERSITY
HAMPTON, VA 23668

ROTE, SUNSHINE MARIE, PHD
ASSOCIATE PROFESSOR
KENT SCHOOL OF SOCIAL WORK
UNIVERSITY OF LOUISVILLE
LOUISVILLE, KY 40292

SCHEIM, AYDEN I, PHD
ASSISTANT PROFESSOR
EPIDEMIOLOGY AND BIOSTATISTICS
SCHOOL OF PUBLIC HEALTH
DREXEL UNIVERSITY
PHILADELPHIA, PA 19104

SHARIFF-MARCO, SALMA, PHD
ASSOCIATE PROFESSOR
DEPARTMENT OF EPIDEMIOLOGY AND BIOSTATISTICS
HELEN DILLER FAMILY COMPREHENSIVE CANCER CENTER
GREATER BAY AREA CANCER REGISTRY (GBACR)
UNIVERSITY OF CALIFORNIA, SAN FRANCISCO
SAN FRANCISCO, CA 94158

SONIK, RAJAN ANTHONY, JD, PHD
DIRECTOR OF RESEARCH
ALTAMED HEALTH SERVICES CORPORATION
LOS ANGELES, CA 90040

TEHRANIFAR, PARISA, DPH
ASSOCIATE PROFESSOR
DEPARTMENT OF EPIDEMIOLOGY
MAILMAN SCHOOL OF PUBLIC HEALTH
COLUMBIA UNIVERSITY
NEW YORK, NY 10032

TOBIN, KARIN E, PHD
ASSOCIATE PROFESSOR
DEPARTMENT OF HEALTH, BEHAVIOR, AND SOCIETY
BLOOMBERG SCHOOL OF PUBLIC HEALTH
JOHNS HOPKINS UNIVERSITY
BALTIMORE, MD 21205

TULU, BENGISU, PHD
PROFESSOR
BUSINESS SCHOOL
WORCESTER POLYTECHNIC INSTITUTE
WORCESTER, MA 01609

VUPPUTURI, SUMA, PHD
SENIOR RESEARCH SCIENTIST
MID-ATLANTIC PERMANENTE RESEARCH INSTITUTE
KAISER PERMANENTE MID-ATLANTIC
ROCKVILLE, MD 20852

WANG, JUNLING NONE, PHD
PROFESSOR AND VICE CHAIR FOR RESEARCH
DEPARTMENT OF CLINICAL PHARMACY
AND TRANSLATIONAL SCIENCE
COLLEGE OF PHARMACY
UNIVERSITY OF TENNESSEE HEALTH SCIENCE CENTER
MEMPHIS, TN 38163

WESCOTT, SIOBHAN M, MD
PROFESSOR & DIRECTOR OF AMERICAN INDIAN HEALTH PROGRAM
COLLEGE OF PUBLIC HEALTH
UNIVERSITY OF NEBRASKA MEDICAL CENTER
GRAND FORKS, ND 58202

WHITT-GLOVER, MELICIA C, PHD
PRESIDENT AND CHIEF EXECUTIVE OFFICER
GRAMERCY RESEARCH GROUP, LLC
ADJUNCT ASSOCIATE PROFESSOR
WAKE FOREST SCHOOL OF MEDICINE
WINSTON-SALEM, NC 27106

WILLIAMS, DONNA L., DRPH
PROFESSOR
DEPARTMENT OF BEHAVIORAL HEALTH SCIENCES
LOUISIANA COMPREHENSIVE CANCER CONTROL PROGRAMS
HEALTH SCIENCE CENTER
LOUISIANA STATE UNIVERSITY, NEW ORLEANS
NEW ORLEANS, LA 70112

WILLIAMS, JONI STROM, MD
ASSOCIATE PROFESSOR
DEPARTMENT OF MEDICINE
DIVISION OF GENERAL INTERNAL MEDICINE
MEDICAL COLLEGE OF WISCONSIN
WAUWATOSA, WI 53226

SCIENTIFIC REVIEW OFFICER

BEHERA, ARUNA K, PHD
SCIENTIFIC REVIEW OFFICER
CENTER FOR SCIENTIFIC REVIEW
NATIONAL INSTITUTES OF HEALTH
BETHESDA, MD 20892

EXTRAMURAL SUPPORT ASSISTANT

ROBINSON, LYNDA K., BS
LEAD GRANTS TECHNICAL ASSISTANT
CENTER FOR SCIENTIFIC REVIEW
NATIONAL INSTITUTES OF HEALTH
BETHESDA, MD 20892

OTHER REVIEW STAFF

FLEMING, LIA CAROLINE, MPH
REVIEW ANALYST
CENTER FOR SCIENTIFIC REVIEW
NATIONAL INSTITUES OF HEALTH
BETHESDA, MD 20892

HONG, SEO YOUNG, MPH
REVIEW ANALYST
CENTER FOR SCIENTIFIC REVIEW
NATIONAL INSTITTUES OF HEALTH
BETHESDA, MD 20892

JAIN, ADITI, BS, MPH
REVIEW ANALYST
CENTER FOR SCIENTIFIC REVIEW
NATIONAL INSTITUTES OF HEALTH
BETHESDA, MD 20892

Consultants are required to absent themselves from the room during the review of any application if their presence would constitute or appear to constitute a conflict of interest.
